# Sulforaphane Synergies with Phytochemicals and Pharmaceuticals: Implications for Healthspan †

**DOI:** 10.3390/medicines13020016

**Published:** 2026-05-06

**Authors:** Jed W. Fahey, Hua Liu

**Affiliations:** 1 Department of Medicine, The Johns Hopkins University School of Medicine, Baltimore, MD 21205, USA; 2 Department of Psychiatry & Behavioral Sciences, The Johns Hopkins University School of Medicine, Baltimore, MD 21205, USA; 3 Department of Physiology, Pharmacology & Therapeutics, The Johns Hopkins University School of Medicine, Baltimore, MD 21205, USA; 4 iMIND Institute, The Johns Hopkins University School of Medicine, Baltimore, MD 21205, USA; 5 Institute of Medicine, University of Maine, Orono, ME 04469, USA; 6 Stanley Division of Developmental Neurovirology, Department of Pediatrics, The Johns Hopkins University School of Medicine, Baltimore, MD 21287, USA; hliu8@jhmi.edu

**Keywords:** cancer, cardioprotection, chemotherapy, HDAC, insulin resistance, neuropathic pain, Nrf2, phytochemical, sulforaphane, synergy

## Abstract

Sulforaphane, a bioactive isothiocyanate found abundantly in cruciferous vegetables, has attracted significant attention for its chemopreventive and therapeutic potential, particularly in cancer. There is now an abundance of peer-reviewed research documenting true synergies between sulforaphane and (a) cancer treatment drugs, (b) pharmaceuticals in development but not yet on the market or in the regulatory pipeline, (c) other phytochemicals, and (d) proprietary mixtures such as leaf extracts and other botanicals, as well as evidence that some cell lines resistant to various cancer drugs become more susceptible when treated with sulforaphane. Most of the published studies demonstrate evidence for synergy in cancer, including cancers of the bladder, blood, brain, breast, colon, esophagus, liver, lung, ovaries, prostate, and skin, where reducing drug dosages could yield substantial patient benefits. Importantly, non-cancer indications have also been reported, such as mitigation of cardiac toxicity, inflammation, obesity, and pain (including antihyperalgesic and antinociceptive effects). Synergistic effects are most often demonstrated in cell line models, with many studies providing robust mechanistic evidence, and some employing the gold-standard Chou–Talalay method for quantifying synergy. Current evidence on the synergistic interactions of sulforaphane with both phytochemicals and pharmaceuticals highlights underlying mechanisms such as modulation of oxidative stress, inflammation, apoptosis, and epigenetic regulation, suggesting significant clinical and therapeutic implications. By providing a comprehensive overview of sulforaphane synergies in both cancer and non-cancer contexts, we aim to inform future research and support the development of integrated therapeutic strategies.

## 1. Introduction

Sulforaphane (SF; 1-isothiocyanato-4-(methylsulfinyl)-butane) is generated when plant myrosinase acts on the glucosinolate glucoraphanin (GR), present at high concentration in broccoli sprouts and other cruciferous vegetables. Over the past three decades, our understanding of SF’s mechanistic actions has evolved considerably. Initially regarded as a promising chemopreventive agent [1], primarily through its induction of phase 2 detoxifying enzymes, SF is now recognized as a multifaceted modulator of numerous cell-signaling and epigenetic pathways relevant to cancer, inflammation, and metabolic regulation [2]. SF has been the subject of over 125 clinical studies (see Supplemental Table S1 [3,4,5,6]. It is now widely regarded as a beneficial component of nutritional support for healthspan and a critical part of the “Food as Medicine” paradigm [7].

While the single-agent benefits of SF have been extensively documented in vitro and in vivo, the recent literature has increasingly highlighted the concept of synergy, where the combined effect of SF and another agent exceeds the sum of their individual effects [8]. There is now an abundance of peer-reviewed research documenting true synergies between SF and (a) cancer treatment drugs, (b) pharmaceuticals in development but not yet on the market or in the regulatory pipeline, (c) other phytochemicals, and (d) proprietary mixtures such as leaf extracts and other botanicals, as well as evidence on cell lines resistant to various cancer drugs that become more susceptible when treated with SF. Almost all of the more than 80 studies addressing SF synergies provide evidence in cell lines or animal models, with many of them utilizing the gold-standard Chou–Talalay method [9,10] to quantify synergy, rather than merely asserting it without quantitative support. Collectively, these studies suggest that synergistic combinations of SF with drugs and other phytochemicals can (a) overcome drug resistance, (b) lower the required therapeutic doses, thus reducing toxicity, and (c) target multiple hallmarks of disease simultaneously [11].

Despite this expanding literature, the field remains fragmented. Reports are dispersed across disease areas, drug classes, phytochemical pairings, and experimental platforms, and they vary substantially in formulation, dosing schedule, mechanistic depth, and rigor of synergy assessment. Some studies focus on chemosensitization or reversal of resistance, whereas others emphasize toxicity mitigation, epigenetic regulation, redox signaling, stemness, or nano-enabled delivery. As a result, it is difficult to determine which findings are broadly reproducible, which are model-specific, and which combinations have the strongest translational rationale. Human evidence also remains limited compared with the large preclinical literature, creating an important gap between mechanistic promise and clinical applicability.

This narrative review was undertaken to address that gap by synthesizing peer-reviewed primary research articles and recent high-quality reviews that examine SF interactions with pharmaceuticals and phytochemicals across cancer and non-cancer indications. It identifies in vitro, in vivo, and clinical reports evaluating SF in combination with chemotherapeutics, targeted agents, investigational drugs, or other bioactives. The emphasis was placed on (1) studies reporting synergy (including Chou–Talalay or similar dose–effect analyses); (2) reports demonstrating mechanistic rationale (e.g., nuclear factor erythroid 2–related factor 2 (Nrf2), histone deacetylases (HDAC)/DNA methyltransferase (DNMT) modulation, apoptosis/autophagy, drug-transport modulation); and (3) translational studies reporting toxicity mitigation or effects observed in animal models or early human trials. Given the extensive nature of the field, it was not feasible to cover every possible combination. Instead, representative and high-impact studies across various tumor types and non-cancer applications were chosen to illustrate recurring patterns and mechanistic themes. Databases utilized included SCOPUS, PubMed, and clinicaltrials.gov.

Phytochemicals like SF are non-patentable and readily accessible through various fruits and vegetables, as well as dietary supplements. Unfortunately, this often results in a lack of funding for clinical trials that could demonstrate their potential to reduce reliance on pharmaceuticals, particularly in the treatment of cancer and other chronic diseases. Consequently, it is unlikely that these phytochemicals will be integrated into FDA-approved Indications for Use and clinical practice guidelines without massive support from practitioners of complementary medicine and integrative health, alongside rigorous clinical studies demonstrating their efficacy in human beings. Most evidence currently available is derived from studies conducted in cell lines and animal models. Therefore, we conclude this paper with an appeal for more clinical trials and reiterate the importance of addressing funding sources for such research.

## 2. Mechanistic Basis for Sulforaphane Synergy

**Figure 1:**
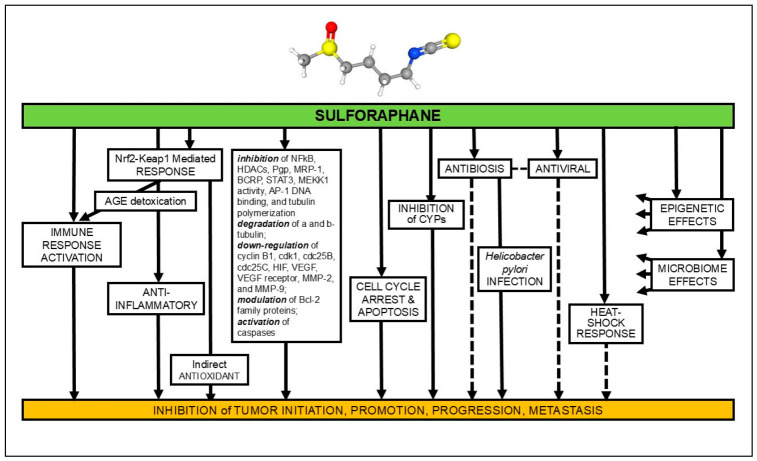
Main anticancer pathways by which sulforaphane has been shown to act.

SF is biologically promiscuous [4,6,12]. Its rapid conversion from the biologically inert precursor GR and subsequent swift uptake by cells throughout the body enables SF to engage multiple pathways simultaneously, facilitating synergistic interactions with other biologically active compounds (Figure 1). The rapid conversion from GR to SF is a partially enzyme-catalyzed process that has been well studied and extensively reported upon [13,14,15,16]. Collectively, these interlocking mechanisms provide a biological foundation for SF’s ability to enhance anticancer therapies while mitigating toxicity in healthy tissues. We enumerate herein a few of them that have been specifically invoked in the examples of synergy discussed in this review.

### 2.1. Nrf2-Mediated Antioxidant and Cytoprotective Signaling

SF is a potent activator of Nrf2 signaling through covalent modification of cysteines on its chaperone protein, Kelch-like ECH-associated protein 1 (Keap1), which stabilizes Nrf2 and promotes the expression of antioxidant and detoxifying genes (e.g., NAD(P)H quinone dehydrogenase 1 (NQO1), heme oxygenase 1 (HMOX1 or HO-1), and enzymes involved in glutathione biosynthesis) [17,18,19,20]. This process enhances the overall antioxidant capacity of cells, providing protection against oxidative stress induced by pharmaceuticals, and potentially improving their efficacy while reducing side effects. These protective effects of SF have been extensively documented in non-malignant tissues, and the activation of the Nrf2 pathway explains many of its beneficial actions. One notable example of SF’s protective effects is its ability to protect cardiomyocytes from damage caused by the chemotherapeutic agent doxorubicin [21]. However, while the upregulation of the Nrf2 pathway by SF enhances protection in healthy tissues, it can also sensitize cancer cells to redox-modulating therapies by altering cellular reactive oxygen species (ROS) buffering and metabolic state, as well as by upregulating pro-apoptotic factors such as microtubule-associated protein 1 light chain 3β (LC3B), cleaved poly(ADP-ribose) polymerase (cPARP), caspases, BAK1, Bid, Bcl-2 Associated X protein (BAX), DNA damage-inducible transcript 3 (CHOP), tumor protein p53, p27, p21, and tumor necrosis factor-alfa (TNF-α), and by downregulating or repressing the expression of factors associated with tumor progression and survival, including nuclear factor-kappa B (NF-κB), interleukin-6 (IL-6), marker of proliferation Kiel 67 (Ki-67), β-catenin, Cyclin K1, cyclin-dependent kinase (CDK), E-cadherin, HDAC, vascular endothelial growth factor (VEGF), sex determining region Y-box2 (SOX2), Notch-1, and B-cell lymphoma 2 (Bcl-2) [11].

### 2.2. Epigenetic Regulation: HDAC and DNMT Inhibition

SF and its metabolites have been shown to exert significant epigenetic effects by inhibiting the activity of HDAC and DNMT, leading to alterations in chromatin structure and gene expression profiles, which can reactivate silenced tumor suppressor genes (e.g., p21, BAX). Epigenetic modulation plays a pivotal role in SF-mediated reprogramming of cancer cell transcription, making it a highly plausible mechanism for synergistic interactions with agents whose efficacy depends on transcriptional context [22,23].

### 2.3. Modulation of Cell Death Pathways: Apoptosis, Autophagy, and Proteostasis

SF plays a crucial role in modulating cell death pathways, including both apoptosis and autophagy. This dual action is particularly significant in the therapeutic context, as SF can sensitize cancer cells to apoptotic stimuli from chemotherapeutics. Specifically, SF influences both intrinsic and extrinsic apoptosis pathways, modulating the expression of pro-apoptotic factors and anti-apoptotic proteins, as well as various caspases that are instrumental in the apoptotic cascade. By shifting the balance toward apoptosis, SF not only enhances the effectiveness of therapeutic interventions but also has the potential to reduce tumor resistance to treatment. Moreover, SF’s activation of autophagy can function as a protective mechanism for normal cells, facilitating the degradation of damaged cellular components and maintaining cellular homeostasis. However, in cancer cells, the effect of SF on autophagy can be context-dependent; it may either inhibit or induce autophagic flux based on dose and specific conditions. This modulation can lead to a paradoxical situation where increased autophagy may sensitize cancer cells to death signals. Evidence also suggests that SF impacts unfolded protein response signaling, which is critical for managing cellular stress. These mechanisms interact with various chemotherapeutics as diverse as 3-methyl adenine (not an approved human drug) and chloroquinone that induce apoptotic stress. Additionally, SF has been found to work synergistically with proteasome inhibitors, tipping the balance toward cell death and contributing to enhanced therapeutic efficacy [24,25,26].

### 2.4. Modulation of Drug Metabolism, Transport, and Resistance

SF can influence cytochrome P450 enzymes (CYPs) and other drug-metabolizing enzymes. By modulating these enzymes, SF can alter the metabolism of pharmaceuticals, potentially enhancing their efficacy or reducing toxicity. SF has been shown to downregulate drug-efflux transporters (e.g., P-glycoprotein) and alter metabolic enzymes relevant to drug resistance, thereby increasing intracellular drug accumulation and potency [11,27].

### 2.5. Modulation of Inflammatory Pathways and Immune Response

Chronic inflammation is a recognized contributor to various diseases, including cancer. SF’s ability to modulate inflammatory pathways further underscores its therapeutic potential. The direct targeting of the critically important NF-κB pro-inflammatory pathway by SF was first laid out by Gerhauser’s group in 2001 [28] and has since been widely studied [29]. By suppressing NF-κB signaling and its downstream inflammatory and survival pathways, SF effectively reduces the production of pro-inflammatory cytokines such as IL-6 and TNF-α, which are implicated in cancer progression and resistance to therapy. As a result, SF enhances tumor cell sensitivity to conventional chemotherapeutic agents and reverses multiple mechanisms of drug resistance [11,30,31,32,33]. Moreover, it is now understood that there is substantial crosstalk between the Nrf2 and the NF-κB pathways, with both co-regulating cellular responses to inflammation and oxidative stress [34].

In addition to its effects on inflammation, SF may also enhance the immune response by modulating immune cell phenotypes, such as promoting an M1 to M2 microglial shift, which has significant implications for the tumor microenvironment [35,36]. Furthermore, SF enhances the activity of natural killer (NK) cells, leading to a more robust cytotoxic immune response against tumors. This immunomodulatory effect is particularly relevant for developing combination therapies that synergistically integrate SF with other immunomodulatory agents [37,38].

### 2.6. Other Effects

Although these effects have yet to be directly linked to mechanisms of synergy with SF, there are a variety of effects that we and others have documented for SF that are likely to contribute to some of the synergies documented herein. These include:

#### 2.6.1. Stimulation of the Heat Shock Response

SF activates the heat shock response, which is important in cancer, neurodegenerative, and neurodevelopmental conditions [6,39,40,41,42]. However, in pancreatic cells SF induced degradation of heat shock protein 90 (Hsp90) client proteins, blocking its interaction with p50 (Cdc37) which serves as a co-chaperone [43].

#### 2.6.2. Selective Antibiosis

SF exhibits selective antibacterial activity, particularly against the carcinogenic bacterium *Helicobacter pylori*, and antiviral potency against SARS-CoV-2 and other coronaviruses [44,45,46,47,48,49,50,51,52,53].

#### 2.6.3. Cell Cycle Arrest

SF induces cell cycle arrest in either the S-phase [54] or G2/M phase [55,56], depending on the type of cancer. By halting cell proliferation, it can make tumor cells more susceptible to the effects of chemotherapy that target dividing cells.

#### 2.6.4. Blood Vessel Dilation and Wound Response

SF promotes blood vessel dilation and modulates wound responses, which are important in cardiovascular disease (CVD) and ischemia–reperfusion injury [57,58,59,60,61,62,63,64,65].

#### 2.6.5. Slow Release of H_2_S from SF

This mechanism is distinct from the more classically appreciated direct N=C=S mediated effects of SF and other isothiocyanates. Thus, H_2_S slow release appears to facilitate: (a) antinociceptive effects—SF promoted the reduction in neuropathic pain induced by a variety of chemotherapeutic agents in tandem with its H_2_S generation [66,67,68]; (b) antihypertensive and vasoprotective activity wherein SF promoted blood vessel dilation (vasodilation) by stimulating endogenous H_2_S production, activating potassium channels in arterioles, improving endothelial function, reducing inflammation, and protecting against vascular dysfunction [65,69]; (c) the cardioprotective activity of SF was first posited by Angeloni’s group [70,71] and has since been shown to occur via a variety of mechanisms (e.g., Nrf2 activation, anti-inflammatory, protection against ischemic injury, improved vascular function, inhibition of platelet aggregation, and anti-atherosclerotic, -fibrotic, and -hypertrophic activity; however, the H_2_S-mediated effects have received much attention in recent years [72], and (d) cognitive function [73].

#### 2.6.6. SF and Cancer Stem Cells (CSCs)

Accumulating evidence indicates that SF targets key regulatory mechanisms that sustain CSC populations. SF suppresses aberrantly activated embryonic signaling pathways, commonly implicated in CSC maintenance, including Sonic Hedgehog (SHH), Wnt/β-catenin, Cripto-1 (CR-1), and Notch. Concomitantly, SF downregulates the expression of multiple CSC-associated markers and transcriptional regulators, such as CD (cluster of differentiation)133, CD44, aldehyde dehydrogenase (ALDH), c-Myc, Nanog (a homeobox protein transcription factor), Oct-4, human telomere reverse transcriptase (hTERT), and matrix metalloproteinase MMP2, while promoting expression of the epithelial marker E-cadherin. Through these coordinated actions, SF reduces CSC stemness, invasiveness, and migratory capacity, as well as diminishes epithelial-to-mesenchymal transition (EMT), ultimately favoring apoptotic cell death. This mode of action presents an attractive target in a number of cancer settings [74,75,76,77].

## 3. Quantitative Synergy: Evidence and Methods

Understanding the quantitative assessment of synergy is crucial for translating preclinical findings into clinical applications. A growing fraction of combination studies utilize formal dose–effect analyses, such as the Chou–Talalay method (combination index, CI), to assess synergy (CI < 1) [9,10]. When applied, SF frequently yields CI values suggesting synergy at pharmacologically relevant concentrations in vitro and additive-to-synergistic effects in vivo [11,78]. Although robust preclinical synergy exists, clinical evidence of improved outcomes remains limited. Factors contributing to this limitation include heterogeneity in SF dosing (e.g., broccoli sprout extracts vs. stabilized SF formulations), differences in pharmacokinetics, and regulatory complexities. To achieve translationally meaningful trials, it is essential to standardize formulations, quantify biomarkers (e.g., Nrf2 target induction), and incorporate validated synergy metrics into trial design.

## 4. Synergies Between Sulforaphane and Pharmaceuticals

Preclinical evidence demonstrates that SF exhibits synergistic effects with various classes of cytotoxic drugs (anthracyclines, platinum agents, taxanes, antimetabolites), targeted agents, pro-apoptotic biologics, and newer experimental agents.

### 4.1. Anthracyclines (e.g., Doxorubicin)

Multiple studies indicate that SF enhances the antitumor activity of doxorubicin (DOX) while also protecting cardiomyocytes from DOX-induced oxidative damage. In breast cancer models, SF has been shown to increase DOX accumulation and promote tumor cell apoptosis. Simultaneously, Nrf2-mediated antioxidant responses provide protection to cardiac tissue in rodent models [21,36,79]. A Phase I/II clinical study has been proposed to investigate the combination of SF or broccoli sprout extract with DOX to mitigate cardiotoxicity (ClinicalTrials.gov NCT03934905) [80].

### 4.2. Platinum Agents (e.g., Cisplatin, Carboplatin)

SF synergizes with cisplatin across multiple cell lines and xenograft models, such as those for squamous cell carcinoma, ovarian and bladder cancers. This synergy reverses resistance phenotypes and diminishes clonogenic survival [81,82]. The underlying mechanisms include the inhibition of DNA repair and survival pathways, alongside enhanced ROS-mediated DNA damage and cancer cell apoptosis.

### 4.3. Taxanes (e.g., Paclitaxel, Docetaxel) and Antimicrotubule Agents

Studies in triple-negative breast cancer (TNBC) models indicate that treatment with taxanes, such as paclitaxel and docetaxel, may paradoxically promote the expansion of cancer stem cells (CSCs), at least in part through the induction of interleukin-6 (IL-6) secretion. In contrast, SF preferentially targets and eliminates CSCs by suppressing NF-κB signaling, including the inhibition of p65 nuclear translocation and the downregulation of p52 and its downstream transcriptional activity. Consistent with these effects, SF effectively reverses taxane-induced enrichment of ALDH–positive cells and significantly reduces both the size and number of primary and secondary mammospheres. Notably, in advanced orthotopic TNBC xenograft models, extreme limiting dilution analysis (ELDA) demonstrates that combined treatment with docetaxel and SF results in greater suppression of primary tumor growth and a significant reduction in secondary tumor formation compared with either agent alone. In TNBC cells, which are known for rapid progression and high relapse rates, SF has demonstrated the ability to sensitize these cells to paclitaxel-induced apoptosis, often via downregulation of NF-κB signaling and destabilization of survival pathways. Additionally, SF-mediated epigenetic remodeling enhances the expression of pro-apoptotic mediators [22,83].

### 4.4. Antimetabolites and Topoisomerase Inhibitors (e.g., 5-Fluorouracil, Topotecan)

Combinations of SF with 5-fluorouracil and other antimetabolites show additive to synergistic effects in colon and other cancer cell types, with SF enhancing apoptotic and cell-cycle effects while sometimes overcoming resistance by influencing metabolic enzymes [3,11].

### 4.5. TRAIL, Proteasome Inhibitors, and Targeted Agents

When tumor necrosis factor–related apoptosis-inducing ligand (TRAIL)-resistant cell lines are treated with SF, it enhances the effects of TRAIL on apoptosis across various cancer models, including rhabdomyosarcoma, bladder, prostate, liver, and osteosarcoma [84,85,86,87,88,89,90]. SF also shows promising synergies with proteasome inhibitors [11,74,91], as well as experimental agents and formulations that target cancer stem cells [76,92,93,94,95,96,97,98,99,100,101,102,103,104,105].

### 4.6. Investigational/Experimental Drugs Paired with Nanoparticle Delivery

Recent studies have explored combinations of SF with established pharmaceuticals, such as the ionophore antibiotic salinomycin, as well as innovative molecules formulated into nanoparticles to improve tumor delivery and synergistic effects [92,105]. It enhanced the anticancer effects of salinomycin in colorectal cancer [92], strengthened solid lipid nanoparticle-based combinations in pancreatic cancer models using aspirin and curcumin [98] or ibuprofen [106], and improved the efficacy of tamoxifen- [107] and DOX-based [108] nanoformulations in breast cancer. Other studies have gone further by embedding SF within the nanoplatform itself, as seen with SF-conjugated selenium nanoparticles [101] and lignin nanoparticles co-loaded with SF and teriflunomide [105]. Together, these studies suggest that SF is not only a bioactive partner in combination therapy but also a useful component of targeted nanomedicine design.

## 5. Synergies Between Sulforaphane and Other Phytochemicals and Botanical Mixtures

Phytochemical co-treatments can produce synergistic effects by acting on complementary pathways, such as the epigenetic modifications by SF combined with the signaling modulation provided by compounds like epigallocatechin-3-gallate (EGCG). However, caution must be observed when utilizing non-standardized botanical extracts which can profoundly introduce the reproducibility of in vitro, in vivo, or clinical trial results. Both deliberate and inadvertent adulteration are common in the industry and are being monitored by the American Botanical Council’s Botanical Adulterants Prevention Program. By design, botanical extracts contain multiple phytochemicals which makes it more difficult to assign cause and effect. Although pure SF or its precursor GR with added myrosinase are most frequently used in in vitro studies, extracts or plant parts containing SF or its precursor GR are almost exclusively used in clinical studies (Supplementary Table S1) for both regulatory and safety reasons.

There are now many examples of SF synergies with other phytochemicals, and a recent review has gone so far as to call SF, curcumin, EGCG, resveratrol, and genistein the “Big Five” phytochemicals targeting cancer stem cells [109]. The mechanistic rationales for phytochemical synergy often converge on critical cancer stem cell pathways, promote epigenetic reprogramming, and inhibit survival signaling. This enables a multi-faceted approach to suppress tumor growth and metastasis while potentially reducing toxicity [76,110,111,112,113].

### 5.1. SF + EGCG/Green Tea Catechins

Multiple studies have reported cooperative anticancer effects of SF and EGCG, particularly in breast and colon cancer models. Their combined treatment enhances apoptosis and suppresses metastatic phenotypes more effectively than either agent alone [114]. Genome-wide epigenetic alterations and a synergistic inhibition of breast cancer cell growth have been documented in vitro, alongside reduced tumor growth in a breast cancer mouse xenograft model following a diet that combines green tea polyphenols and broccoli sprouts [115].

### 5.2. SF + Curcumin/Resveratrol/Genistein

Combinations of SF with curcumin and resveratrol, both of which serve as pleiotropic modulators of NF-κB, signal transducer and activator of transcription 3 (STAT3), sirtuin (SIRT) pathways, often yield additive or synergistic anti-proliferative effects, supported by substantial evidence of complementary epigenetic and anti-inflammatory actions. Similarly, genistein (GE) has demonstrated synergistic effects with SF across various breast cancer models [116,117].

### 5.3. Synergy Within Botanical Extracts and Multi-Component Nutraceuticals

Whole extracts (broccoli sprout extracts, leaf or seed extracts containing SF precursors and co-factors) can exhibit synergy due to the combined effects of SF with other bioactives (chlorophyll derivatives, flavonoids) that modulate absorption, metabolism, or complementary signaling [118]. A noteworthy recent study conducted by researchers at the University of Alabama at Birmingham explored the synergy of phytochemical-rich plant extracts [119]. In their investigation, estrogen receptor-negative transgenic mice were fed diets rich in SF-rich broccoli sprouts, withaferin A-rich ashwagandha, or a combination of the two, compared to control diets. Among other findings, the study reported increased expression of cancer-related p53, p57, (a cyclin-dependent kinase (CDK) inhibitor), BAX, BAX:Bcl-2 ratios, and p53-up-regulated modulator of apoptosis (PUMA). Their epigenetic analysis identified reduced expression of HDAC1 and DNMT3A and elevated expression of HDAC3. Additionally, they observed increased mRNA levels of Sal-like 1 (SALL1) and netrin 4 (NTN4), reduced tumor incidence and extended tumor latency, and changes in microbiome diversity and balance. Interestingly, as detailed and well documented as this study was, they did not report actual levels of SF or withaferin A in the diets or in the input botanicals [119].

## 6. Evidence for Sulforaphane Synergy Across Cancer Types: Representative Examples

**Figure 2:**
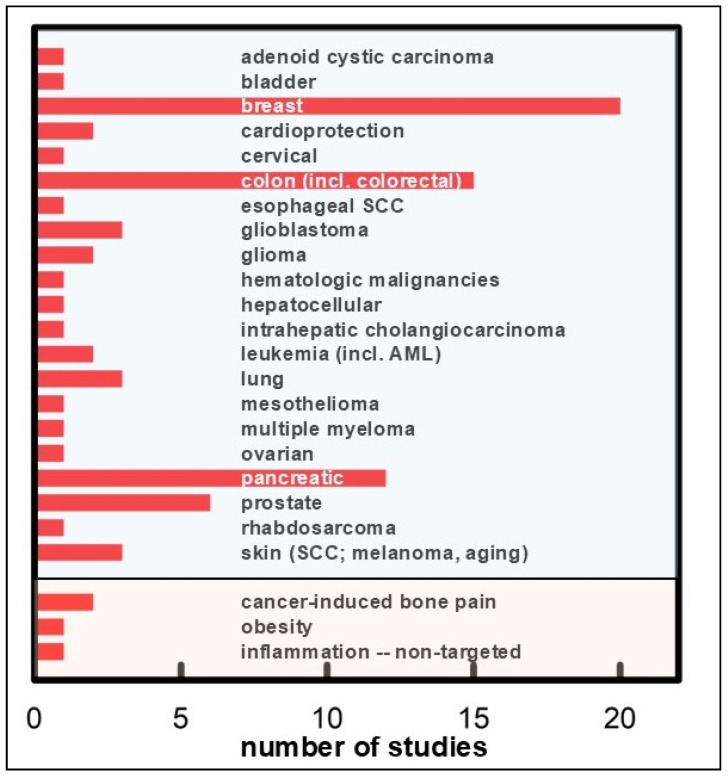
Studies (see Table 1) describing synergy of sulforaphane: with cancer treatment drugs, by cancer type (above horizontal bar; shaded blue), and with non-cancer conditions (below horizontal line; shaded tan).

Illustrative findings follow, organized by tumor type and highlighting reproducible patterns rather than providing a recitation. Table 1 lists synergies categorized by chronic condition (e.g., cancer and others), and Figure 2 highlights the most well-documented cases of SF synergies.

### 6.1. Bladder

The standard of care for metastatic bladder cancer has traditionally involved platinum-based chemotherapy. However, more recent advances have introduced immune checkpoint inhibitors and antibody-drug conjugates, both of which have demonstrated significant benefits. The synergy between these checkpoint inhibitors and platinum-based chemotherapy has been widely studied in a variety of systems [169].

The direct anticancer effects of SF on bladder cancer stem cells involve multiple signaling pathways, including phosphatidylinositol 3-kinase (PI3K), protein kinase B (Akt), mechanistic target of rapamycin (mTOR), mitogen-activated protein kinase (MAPK), NF-κB, STAT3, IL-6 receptor, micro-RNA (miR)-124, Nrf2, zonula occludens-1 (ZO-1), and β-catenin [170]. SF has also been shown to suppress the growth of drug-resistant bladder cancer cell lines while enhancing their sensitivity to platinum-based treatments, resulting in decreased adhesion and invasiveness [27].

Additionally, bladder tumors have also been treated using TRAIL, which promotes apoptosis in various cancer cells but not normal cells. However, in vitro studies indicate that up to 50% of cancers can develop resistance to TRAIL via activation of well-known pathways. Notably, SF has been found to enhance TRAIL-mediated apoptosis in bladder cancer [86], as well as in osteosarcoma, rhabdosarcoma, prostate cancer, and lung cancer [84,85,87,88,89,90].

### 6.2. Blood (Hematologic Malignancies)

CAR-T (chimeric antigen receptor cell therapy) has been widely used to treat hematologic malignancies [171]. The direct and selective effects of SF on tumor cells and immune responsiveness are well established, although its impact on the tumor microenvironment has only recently begun to be explored [35]. Shen and colleagues [147] demonstrated that SF downregulated the expression of the negative immune regulator PD-1 (related to T-cell exhaustion) in CAR-T cells by inhibiting the PI3K/Akt pathway. Additionally, SF promoted PD-L1 degradation in tumor cells through the activation of the ubiquitin-mediated proteolysis pathway, thereby enhancing CAR-T cell antitumor activity in vitro, as well as in a mouse model and in humans. In a Chinese study involving eight patients with relapsed or refractory B-cell hematologic malignancies undergoing CAR-T therapy, they were randomized to receive a placebo or oral commercial broccoli sprout extract for 30 days. Compared to the control group, patients in the group receiving SF alongside CAR-T treatment exhibited lower blood levels of PD-1 and higher expression of proinflammatory cytokines IFN-γ and IL-2 [147].

The ATPase p97 (volosin-containing protein) is a crucial component of the protein homeostasis regulatory network and has been a target in anticancer drug development. The investigational drug CB-5083 is a selective ATP-competitive inhibitor of p97, focused on applications in acute myeloid leukemia and other malignancies. Resistance to CB-5083 often arises through the activation of the NF-κB pathway, which has been shown to be inhibited by SF. Wang and colleagues [120] explored this by combining SF with CB-5083 in vitro and found that SF itself reduced the proliferation of CB-5083 resistant cells alone, while demonstrating a synergistic effect in combination with CB-5083.

SF has also been shown to synergize with the tyrosine kinase inhibitor imatinib (Gleevec) to overcome resistance in imatinib-resistant leukemia stem cells [150]. This combination treatment effectively eliminated CD34^+^/CD38^−^ stem cells, induced levels of intracellular ROS, increased apoptosis with elevated expression of caspase 3, PARP and BAX, while decreasing Bcl-2 expression.

### 6.3. Brain

SF is able to cross the blood–brain barrier [6]. Glioblastoma multiforme is the most aggressive and prevalent primary brain tumor with a very poor prognosis in most cases. SF has been shown to sensitize glioblastoma cells in vitro, causing cell cycle arrest, caspase 3/7 activation, DNA fragmentation, and apoptosis [54].

PNA-a15b is a peptide nucleic acid interfering with the micro-RNA (miR-15b-5p), which has been implicated in the pathogenesis of aggressive gliomas such as glioblastoma. The interference increases apoptotic effects, among other mechanisms. In this context, it has been demonstrated that the combination of SF and PNA-a15b acts synergistically to induce apoptosis in glioblastoma multiforme cells [146] as well as in human colon cancer cells [135].

Moreover, SF has been found to reverse the resistance of glioblastoma cell lines to temozolomide, a DNA alkylating agent used to treat certain glioblastomas and anaplastic astrocytomas. SF significantly suppressed the proliferation and survival capacity of these resistant cell lines. Moreover, it markedly inhibited cell growth and enhanced cell death in a mouse xenograft model derived from temozolomide-resistant cell lines [30].

### 6.4. Breast

Since the pioneering work of the Talalay group at Johns Hopkins, SF has been recognized as a potential tool for the prevention and treatment of breast cancer [1,172]. Various mechanisms have been identified through in vitro and preclinical work over the years, including, but not limited to, inhibition of cancer stem cells, induction of apoptosis, blockage of cancer cell growth and proliferation, suppression of metastasis, modification of estrogen metabolism, alterations in gene expression and DNA methylation, as well as reductions in oxidative stress and inflammation [173].

Notably, numerous in vitro and animal model studies over the past 15 years have documented synergistic effects between SF and both pharmaceuticals and phytochemicals in the context of breast cancer treatment. While many of these have been reviewed recently [8,12], we will highlight a few notable examples herein.

#### 6.4.1. Pharmaceuticals

To our knowledge, one of the earliest studies demonstrating synergy between SF and a breast cancer treatment was conducted by one of us (HL) using exemestane. This drug is a potent inhibitor of aromatase, which converts androgens to estrogens, and is still widely used to treat estrogen-driven breast cancer. SF potently synergizes exemestane in some of its modes of action, thus suggesting that the dose of the drug could be reduced and that SF could have a central role in preventive strategies [126]. However, clinical trials following these observations have yet to be initiated.

SF has been shown to sensitize breast cancer cells to DOX and paclitaxel, while also targeting cancer stem cell phenotypes through epigenetic mechanisms, as demonstrated by Bose et al. [79] and Chan et al. [76]. In TNBC models, combining SF with the taxane docetaxel resulted in reduced primary tumor volume and secondary tumor formation compared to each treatment alone, allowing for lower taxane dosage requirements in a xenograft model [75].

Additionally, Pogorzelska et al. [108] examined the effects of combining SF with the anthracycline DOX in a TNBC animal model. DOX, an established chemotherapy drug, arrests or slows cancer cell growth by blocking the enzyme topoisomerase 2. The synergistic combination of SF and DOX not only reduced primary mammary tumors in a mouse model but also increased nuclear accumulation of DOX while providing cardio-, nephro- and hepato-protective effects.

EMT facilitates the acquisition of mesenchymal characteristics by cancer cells of epithelial origin, leading to disease progression and drug resistance. The use of getifinib has been associated with the development of EMT as a mechanism for cancer cells to escape drug toxicity. In a TNBC cell line, an SF-cisplatin combination inhibited stemness (reversion to pluripotent stem call characteristics) and metastatic potential by down-regulating SIRT-mediated EMT signaling, demonstrating a strong synergistic and multifunctional effect [130].

Furthermore, SF has been shown to work synergistically with 5-fluorouracil, which interferes with the incorporation of pyrimidines in rapidly growing cancer cells, particularly in a highly invasive TNBC cell line. This combination induces autophagy and premature senescence [131,174].

#### 6.4.2. Phytochemicals

Recent investigations have highlighted synergy between SF and biochanin A (BCA), an isoflavone found in *Trifolium pratense* (red clover), as described in a 2024 study [129]. This combination of two phytochemicals suppresses cancer progression in an in vivo breast cancer model by reducing cell proliferation and promoting apoptosis, leading to cell cycle arrest and downregulation of extracellular signal-regulated kinase (ERK)-1/2 and other cell proliferation factors.

Epigenetic mechanisms, specifically DNMT inhibition and HDAC inhibition, were synergistically targeted in breast cancer cells by a combination of phytochemicals and sodium butyrate (NaB) [117]. GE, derived from soybeans and other vegetables, is a potent inhibitor of DNMTs. NaB, a short-chain fatty acid produced by gut bacteria, possesses various antineoplastic properties, including acting as an HDAC inhibitor. Among its many other mechanisms, SF is an HDAC inhibitor. Remarkably, the triple combination of SF, GE, and NaB exhibited the greatest impact, followed by the combinations of SF and GE, SF and NaB, and GE and NaB. These combinations proved to be more effective in suppressing cell proliferation, promoting apoptosis, and inducing cell cycle arrest compared to individual compounds or untreated controls. Furthermore, these combinations enhanced the downregulation of key players in DNA methylation, histone deacetylation, histone methylation, and histone acetylation [117].

### 6.5. Colon

The impact of SF on neoplasia in colon cancer cells is well-documented, with a recent review highlighting 26 individual studies spanning a wide range of cell lines and methodologies, as well as a comprehensive list of mechanisms involved [3].

Among the more mechanistically intriguing pharmaceutical-SF synergies is that between SF and the antibiotic ionophone salinomycin. Isolated from *Streptomyces* spp., salinomycin has been used in veterinary medicine for decades. Over a decade ago it was found to be 100 times more potent than paclitaxel against breast cancer stem cells and effective against many other types of cancer. Liu and colleagues [92] demonstrated that SF and salinomycin synergistically inhibited proliferation, induced apoptosis, and decreased migration and invasion of human colorectal adenocarcinoma cells.

From a dietary perspective, although not a traditional phytochemical, the mineral selenium is a critical part of selenoproteins and is found in cruciferous vegetables alongside SF. SF inhibits carcinogenesis in colorectal cancer cells through multiple mechanisms, including apoptosis induction, proliferation suppression, and enhancement of antioxidant defense. Notably, SF synergizes with selenium by significantly increasing the expression of selenoproteins such as glutathione peroxidase 2 (GPx2) and thioredoxin reductase 1 (TrxR1), surpassing the effects of either compound alone [138]. Recent findings by this research team also demonstrated that the combination of selenium and SF offers synergistical protection to normal colonic epithelial cells against free radical-mediated oxidative damage and cell death [137].

SF also suppresses cyclooxygenase-2 (COX-2)/Akt/glycogen synthase kinase-3 beta (GSK3β) signal transduction, upregulates the expression of caspase-3, and downregulates survivin expression, as does cisplatin [143]. The combined inhibitory effect of cisplatin and SF on survival rates of colorectal cancer cells was found to be more pronounced than that of either agent alone [143].

A triple combination of SF, curcumin, and dihydrocaffeic acid (a metabolite of the phytochemical chlorogenic acid produced by the gut microflora) was evaluated for its selective cytotoxicity against colon cancer cells [78]. Using the Chou–Talalay equation, the research determined that an equimolar mixture of SF and dihydrocaffeic acid exhibits pronounced synergism. In another 3-phytochemical in vitro colon cancer model, the combination of SF, quercetin, and curcumin demonstrated additive, but not synergistic, inhibitory effects on colon cancer cell proliferation [136]. Furthermore, the combination of these 3 phytochemicals enhanced the antiproliferative efficacy of both 5-fluorouracil and cisplatin, two widely used chemotherapy agents.

Kong’s group at Rutgers investigated the effects of combining dibenzoylmethane (DBM), derived from licorice, with SF in an APC^min/+^ model of familial adenomatous polyposis (FAP) [142]. This combination inhibited intestinal adenoma development by 57% and effectively blocked colon tumor formation, while also reducing levels of prostaglandin E2 and leukotriene B4, along with inhibiting key biomarkers such as COX-2, proliferating cell nuclear antigen, cleaved caspases, cyclin D1, and p21.

The platinum-based oxaliplatin disrupts DNA replication and transcription in cancer cells by forming intra-strand DNA adducts. The combination of oxaliplatin and SF has been found to synergistically enhance the inhibition of colonic Caco-2 cell growth, along with changes in associated biomarkers, suggesting that SF sensitizes colon cancer cells to oxaliplatin’s growth-inhibitory effects by activating various modes of cell death, including both extrinsic and intrinsic apoptotic pathways [139].

### 6.6. Gastric

SF has been extensively studied for its effects on gastric cancer and ulcers from two different perspectives. The first approach focuses on the quite potent and selective activity of SF against *Helicobacter pylori*, a major contributor to gastric cancers and classified as a Group 1 carcinogen by the WHO. This has been evaluated through in vitro and in vivo studies, as well as a clinical trial conducted in Japan, where the incidence of both *H. pylori* infection and stomach cancer has historically been very high [44,45,46,47,48]. Although this clinical trial demonstrated a modest effect, SF did not eradicate the bacterium in any of the patients. Therefore, it may serve as a valuable adjuvant to standard antibiotic regimens such as “triple therapy” or “quadruple therapy”, as well as a potential maintenance therapy for individuals at high risk of infection due to occupational and geographical factors [48,175]. The second approach has been to explore the efficacy of SF through a variety of mechanisms in cancer cells [176,177,178,179]. Moreover, a study explored the effects of SF and thymoquinone (TQ) in a rodent model of aspirin-induced gastric ulcer. The results indicated that both compounds effectively reduced gastric ulcer indices, oxidative stress, inflammation, and apoptosis, suggesting their potential as therapeutic agents to mitigate gastrointestinal side effects associated with nonsteroidal anti-inflammatory drug use [179].

### 6.7. Esophageal

Torkinib (PP242), a selective mTOR inhibitor, is used to induce mitophagy and apoptosis selectively in cancer cells. Lu and colleagues [144] investigated its use in conjunction with SF and found that this combination synergistically enhanced anti-tumor activity in esophageal squamous cell carcinoma through the established mechanisms of SF’s action, particularly its regulatory effects on the PI3K/Akt/mTOR pathway.

### 6.8. Liver

Gemcitabine (GEM) is a first-line chemotherapy agent for unresectable intrahepatic cholangiocarcinoma (iCCA), the second most common hepatic malignancy, which is associated with a poor prognosis due to early local invasion, metastasis to the liver and lymph nodes, and a low rate of early diagnoses. Tomooka et al. [149] demonstrated that SF augmented the inhibitory effects of GEM on iCCA growth through multiple mechanisms. Key among these was SF’s potent HDAC inhibitory activity, leading to cell cycle arrest, apoptosis, and suppression of invasion, migration, EMT, and angiogenesis from the combination.

TrxR is a key oxidative stress regulatory enzyme which is overexpressed in various human cancer cells. The effects of auranofin, a TrxR specific inhibitor, on apoptosis in hepatocellular carcinoma cells were enhanced by SF [148]. The combined treatment increased mitochondrial dysfunction and ROS accumulation, while also decreasing TrxR activity in the cancer cells.

### 6.9. Lung

SF has been shown to inhibit the proliferation of lung cancer cells, promote apoptosis, and enhance the effects of cisplatin and targeted therapies in models of non-small cell lung cancer (NSCLC) [180].

Furthermore, SF has shown a synergistic effect in chemosensitizing malignant mesothelioma cells to cisplatin therapy [154]. This synergism appears to be mediated through proapoptotic pathways and cell-cycle modulators. Notably, the inhibition of autophagy induced by bafilomycin A1 (a macrolide antibiotic from *Streptomyces* spp.) was augmented by the cytotoxic effects of the SF-cisplatin combination.

### 6.10. Ovarian

Synergies of SF against ovarian cancer cells have been demonstrated by many groups: (a) SF acts synergistically with EGCG, a catechin polyphenol found in green tea and various fruits, nuts and berries, enhancing its anti-cancer effects in ovarian cancer cells [181]. (b) SF enhances cisplatin-mediated apoptosis, promotes cell cycle arrest, and upregulates the cell cycle inhibitor p21 in ovarian cancer cells [182]. (c) In a mouse xenograft model, SF increases the sensitivity of ovarian carcinomas to cisplatin by upregulating the cancer suppressor miR-30a, leading to increased intracellular cisplatin accumulation and subsequent DNA damage [183]. (d) SF induces growth arrest and apoptosis in epithelial ovarian cancer cells by inhibiting retinoblastoma protein (RB) phosphorylation and enhancing the stability of the RB-E2F-1 complex. Notably, combining SF with the chemotherapeutic agent paclitaxel results in additive growth suppression [184]. (e) Additionally, SF has been shown to synergize with cisplatin to suppress ovarian cancer cell proliferation and promote apoptosis. This study also demonstrated that SF effectively suppressed tumor growth and inhibited the proliferation of human ovarian cancer cell xenografts in nude mice.

### 6.11. Pancreatic

SF has been shown to protect against pancreatic acinar cell injury by modulating Nrf2-mediated oxidative stress markers (e.g., GPx, superoxide dismutase (SOD), malondialdehyde), and by suppressing activation of the nucleotide-binding domain, leucine-rich-containing family, pyrin domain-containing-3 (NLRP3) inflammatory pathway [185].

Various combinations of chemotherapeutic drugs with SF have also been explored in pancreatic cancer preclinical studies. Early research on SF’s synergistic effects in pancreatic cancer indicated that SF modulated proliferation and apoptotic pathways, demonstrating an additive effect when combined with gemcitabine in preclinical models [156].

Thakkar and colleagues at Ohio State [159] further evaluated the effects of a combination of aspirin (acetylsalicylic acid), curcumin, and SF on multiple pancreatic cell lines. They found that at concentrations 2.5- to 8-fold lower than those required for each agent alone, the combination (1 mM aspirin, 10 µM curcumin, and 5 µM SF) significantly reduced cell survival by 70%, increased apoptosis by 51%, activated caspase-3, and inhibited NF-κB DNA binding by 45–75%. Furthermore, this combination promoted the expression of P-ERK1/2, c-Jun, p38, MAPK, and p53, suggesting sustained activation of the ERK1/2 signaling pathway as one of the possible mechanisms behind its efficacy.

In an effort to enhance drug delivery to pancreatic cancer patients, Desai and colleagues [162,186] developed a self-emulsifying drug delivery system that encapsulates both SF and loratadine. They reported a 40-fold reduction in the IC_50_ for the SF-loratadine combination compared to loratadine alone, with an additional 10-fold decrease in IC_50_ for the nanoformulation in pancreatic cancer cell lines. This advancement allowed for much lower doses of loratadine, although this approach has yet to progress to clinical settings.

Sorafenib, a kinase inhibitor approved for human use in renal, hepatocellular, and thyroid cancers, was evaluated for its effects on pancreatic cancer both in vitro and in vivo [160]. Furthermore, the combination of sorafenib and SF exhibited synergistic effects, markedly enhanced the elimination of pancreatic cancer stem cell characteristics compared to either compound alone. This combination also eradicated sorafenib-induced NF-κB binding, which was linked to abrogated clonogenicity, spheroid formation, ALDH1 activity, migratory capacity, and increased apoptosis. In a mouse xenograft model, the combination of SF and sorafenib synergistically reduced tumor size through mechanisms involving apoptosis induction, proliferation inhibition, angiogenesis suppression, and downregulation of some of the undesirable protein expression outcomes associated with sorafenib treatment [160].

The German placebo-controlled POUDER trial investigated the effects of a freeze-dried broccoli sprout intervention (15 capsules/day) in patients with advanced pancreatic ductal adenocarcinoma (*n* = 29), comparing it to a placebo group (*n* = 11) over one year [187,188]. Despite high drop-out rates (primarily due to patient’s unwillingness to consume the required number of capsules), and increased mortality, the data displayed a non-significant effect of treatment after six months. By 360 days, only 8 and 5 patients (treatment and control, respectively) remained. Notably, the glucoraphanin dose in this study was relatively high (analytical figures provided were 0.51 µmol SF plus 411 µmol glucoraphanin per day) in contrast to other studies utilizing broccoli phytochemicals [5].

A variety of triple combinations of compounds have also been evaluated with both malignant and non-malignant pancreatic ductal cell lines. Appari et al. utilized combinations of SF with green tea catechins (e.g., EGCG) and quercetin, reporting reduced viability, migration, expression of MMP-2, MMP-9, and ALDH1 activity, spheroid formation and induced apoptosis as well as effects on K-ras inhibition [161]. Sutaria and colleagues have developed solid lipid nanoparticle delivery systems to test a combination of SF (5 µM) with aspirin (25 µM) and curcumin (2.5 µM) against pancreatic cancer cell lines. They reported favorably upon pharmacokinetics, stability, shelf life, targeting parameters and apoptosis-related outcomes [98].

### 6.12. Prostate

SF has demonstrated widespread in vitro efficacy and has shown a modest but measurable effect in several clinical trials involving prostate cancer [189,190,191,192]. Additionally, SF inhibits HDAC activity, reactivates tumor suppressors, and enhances the effectiveness of androgen-deprivation strategies [12,23,192,193,194,195,196,197,198,199]. Combinations with other phytochemicals (e.g., genistein) produce additive anti-proliferative effects [22].

Certain prostate cancer cells are initially responsive to androgen deprivation therapy but can eventually develop resistance, resulting in a state known as “castration-resistant” prostate cancer. These patients may then be treated with 2nd generation anti-androgens such as enzalutamide. Research by Khurana and colleagues [163,164] indicates that at micromolar doses, SF may serve as an effective adjunct to current treatment protocols for patients with castration-resistant prostate cancer. The underlying molecular mechanisms involve repression of Hsp90, inhibition of HDAC, and upregulation of Nrf2 [22,196]. Furthermore, the Khurana group found that SF sensitized castration-resistant prostate cancer cells to enzalutamide, and augmented the effects of ganetespib (a potent experimental antiangiogenic and Hsp90 inhibitor) [164].

Investigations involving stem cell-enriched prostate cancer cell lines by the Kallifatidis group have illustrated mechanisms of synergy between SF and both taxol and cisplatin [156]. Their experiments revealed that combinations of SF with either taxol or cisplatin completely abrogated clonogenicity and dramatically increased apoptosis compared to either treatment alone, with no toxicity observed in nonmalignant cells.

The investigational drug TRAIL specifically targets malignant cells, sparing normal cells, and has been extensively studied over the years. Shankar and colleagues found that SF enhanced the therapeutic potential of TRAIL against prostate cancer cells, sensitized TRAIL-resistant cells, induced apoptosis, and enhanced the antitumor activity of TRAIL in an orthotopic mouse model. They also noted that the combination resulted in enhanced expression of pro-apoptotic proteins BAX and BAK, and reduced expression of anti-apoptotic proteins Bcl-2 and Bcl-XL_._ Additionally, it inhibited various MMPs, hypoxia-inducible factor (HIF)-1α, COX-2, and regulated FOXO3a more effectively than treatment with either agent alone [90]. Labsch et al. treated advanced androgen-independent prostate cancer cells enriched with apoptosis-resistant cancer stem cells using a combination of TRAIL and SF [88]. This combination produced a stronger effect than either agent alone, demonstrating significant inhibition in in vivo tumor engraftment and growth studies. They attributed this strong inhibition to an SF-induced shift from TRAIL-mediated survival signals in resistant cells to apoptosis.

### 6.13. Skin

Epidermal squamous cell carcinoma is often treated with cisplatin. Studies have shown that squamous cell carcinoma cell lines treated with both cisplatin and SF exhibited a reduced number of cancer stem cells across various models. Their combination demonstrated increased responsiveness to treatment and significantly diminished tumor growth, as evidenced by a reduction in tumor volume in “spheroid” models [165].

Studying cultured melanoma cells, researchers found that a combination of the flavonoid phytochemical quercetin and SF, delivered by injection proximal to the tumor site, inhibited cell proliferation and migration more effectively than either compound alone. In a mouse model, this combination suppressed melanoma growth, which was attributed to reduced MMP-9 expression in the tumors [167].

Another noteworthy example of phytochemical combinations involved SF and a proprietary ingredient in both commercial topical and oral products known as Fernblock^®^. This ingredient is derived from the fern *Polypodium leucotomos* and contains *p*-coumaric, ferulic, caffeic, vanillic, and chlorogenic acids [166]. In vitro studies with melanoma cells using this combination demonstrated a true synergistic effect on the inhibition of cancer cell migration and the production of MMP-1, -2, -3, and -9, as well as inflammasome activation and interleukin-1 beta (IL-1β) secretion. Additionally, the researchers showed that in normal immortalized skin cells, the combination resulted in synergistic production of inflammation (TNF-α)-induced MMPs, alongside enhanced antioxidant activity.

## 7. Non-Cancer Synergies and Translational Opportunities

The synergy of SF extends beyond oncology, encompassing several non-malignant conditions that warrant attention. Refer to Table 1 for a comprehensive list of synergies categorized by chronic condition.

### 7.1. Cardioprotection (Attenuation of Anthracycline Cardiotoxicity)

DOX is widely used in the treatment of early-stage, node-positive, and metastatic breast cancer, particularly in patients with human epidermal growth factor receptor-2 (HER2)-positive tumors. Research conducted by Singh’s group at Texas Tech has demonstrated that SF protects heart myoblasts from DOX-induced damage and toxicity [200]. Their studies further revealed that SF works synergistically with DOX to promote cancer regression while also safeguarding the heart from the drug’s toxicity in preclinical models [21,79]. It was shown that SF activated Nrf2 in cardiac tissue, reduced mitochondrial ROS, and preserved contractile function during DOX exposure, while enhancing the sensitivity of tumors to DOX [21,79]. Currently, this research group is engaged in a clinical trial to investigate whether these cardioprotective effects of SF create a valuable therapeutic window for combination therapy aimed at reducing anthracycline cardiotoxicity [80].

### 7.2. Neuroinflammation and Neuropathic Pain

Cancer-induced bone pain (CIBP) is characterized by persistent pain, spontaneous intermittent pain, and abrupt pain triggered by activity, significantly impacting the quality of life for cancer patients. The clinical management of CIBP often involves the administration of nonsteroidal anti-inflammatory drugs (NSAIDs), opioids, and various adjuvant therapies such as radiation therapy, surgery, chemotherapy, and antiepileptic drugs.

A recent study [132] has provided compelling evidence for the anti-nociceptive effects of SF through various mechanisms, including the enhancement of the antihyperalgesic effects of morphine. Although the mechanisms are not fully understood, SF appears to promote the expression of a key opioid receptor. Fu et al. [132] also investigated the impact of intrathecal injection of SF (administered into the spinal canal or the subarachnoid space) on modulating CIBP and augmenting the analgesic effects of morphine. In their rat cancer model, SF demonstrated several beneficial effects: (a) alleviation of painful behavioral hypersensitivity, (b) activation of Nrf2 and HO-1, (c) inhibition of NF-κB, TNF-α, IL-1β, IL-6, and inducible nitric oxide synthase (iNOS), (d) suppression of the proliferation of allograft breast cancer cells injected into the bone marrow, (e) promotion of mu-opioid receptor (MOR) expression in vitro in a human neuroblastoma cell line, and (f) enhancement of the antihyperalgesic effects of morphine in CIBP rats by restoring the downregulation of the MOR expression in the spinal cord [132].

These findings underscore the potential role of SF as a valuable adjunct in the management of CIBP, highlighting its ability to address both pain and underlying mechanisms of neuroinflammation. Additionally, SF has been shown to reduce microglial NLRP3 inflammasome activation, promote anti-inflammatory microglial phenotypes, and decrease neuropathic pain behaviors in rodent models. Co-administration with opioids (e.g., morphine) can further potentiate analgesia while attenuating tolerance and hyperalgesia in preclinical studies [38,201,202]. These interactions implicate Nrf2-mediated anti-inflammatory and antioxidant pathways as key mediators in the therapeutic effects of SF.

### 7.3. Metabolic Disease and Obesity

Using adipocytes to model obesity, the omnipresent antioxidant, anti-inflammatory, and anticancer phytochemical flavonoid myricetin, when combined with SF, synergistically and potently induced apoptosis in adipocytes [155].

SF has also been shown to inhibit hepatic gluconeogenesis, improve glucose tolerance, and enhance insulin sensitivity in animal models. Additionally, small human trials using broccoli sprout extract have demonstrated modest improvements in glycemic parameters [203,204,205]. The potential for synergy between SF and antidiabetic agents (e.g., metformin) has been explored in preclinical studies, suggesting that SF may enhance AMPK/Nrf2 axis signaling and thereby amplify metabolic benefits [102,206].

### 7.4. Anti-Inflammatory and Organ-Protective Effects

Luteolin, an anti-inflammatory flavone that is widely distributed in various vegetables (including broccoli), acts as a direct antioxidant. Rakariyatham and colleagues [168] demonstrated a synergistic attenuation of cellular oxidative stress when SF was combined with luteolin in a widely used mouse macrophage-like tumor cell line.

SF has also been shown to mitigate various toxicities, including nephrotoxicity and hepatotoxicity, induced by chemotherapeutics and other toxins in animal models. Preclinical studies have shown that SF attenuates hepatic injury caused by agents such as acetaminophen, sodium valproate, cisplatin, and other hepatotoxins through Nrf2-driven antioxidant/detoxification responses and preservation of mitochondrial redox homeostasis [207,208,209,210,211,212,213,214,215]. However, SF is better described as an adjunctive protective co-intervention rather than a formally validated synergistic partner, because the available data mainly show a reduction in drug-induced toxicity rather than direct enhancement of therapeutic efficacy [216,217,218].

## 8. Pharmacokinetic, Formulation, and Dosing Considerations

The clinical translation of SF synergy depends on addressing pharmacokinetic (PK) and pharmacodynamic (PD) variability:

- **Formulation variability.** SF can be administered as broccoli sprout or seed extracts (BSE), stabilized SF preparations, or glucoraphanin with or without active myrosinase. Bioavailability can vary significantly depending on the formulation, the activity of co-ingested myrosinase, and the composition of the gut microbiome [4,203,204].
- **Dose and timing.** Many preclinical studies use SF concentrations that are challenging to achieve in humans with dietary intake alone. Optimizing the timing of SF administration relative to pharmaceuticals (e.g., whether as pretreatment to induce epigenetic changes or concurrent dosing to enhance drug uptake) will be crucial.
- **Biomarkers.** Reliable biomarkers of SF exposure (e.g., plasma SF metabolites, induction of NQO1 or HO-1 in peripheral blood mononuclear cells) and PD readouts (HDAC activity assays, Nrf2 target induction) should be integrated into trial designs to confirm target engagement.
- **Safety.** Human studies involving BSE and SF formulations have reported excellent tolerability and safety at moderate doses, with gastrointestinal side effects being the most common. However, the long-term safety of a specific combination with cytotoxic agents requires systematic evaluation [203,219].

## 9. Quality of Evidence, Limitations, and Risks

### 9.1. Preclinical Predominance and Translational Gaps

Most synergy data currently available is derived from in vitro and animal studies. While many of these studies exhibit robust mechanistic insights, their predictive value for human efficacy remains uncertain without standardized formulations and validated biomarkers [11].

### 9.2. Context-Specific Effects and Potential Antagonism

The pleiotropic nature of SF raises the possibility of context-dependent antagonism. For example, Nrf2 activation can promote cytoprotection and chemoresistance in some tumors if activated in tumor cells rather than in the surrounding stroma. It is essential to carefully assess tumor Nrf2 status and to develop dose/timing strategies that preferentially protect normal tissues without rescuing tumor cells [220,221,222]. If Nrf2 is constitutively upregulated, then additional administration may not yield further effects. Notably, fewer than 1% of cancers listed on The Cancer Genome Atlas (TCGA) website exhibit mutations in either Nrf2 or Keap1 [223].

### 9.3. Methodological Heterogeneity

Heterogeneity in synergy assessment, inconsistent application of formal combination analysis, and variable reporting standards impede the potential for meta-analytic synthesis. Future studies should employ standardized combinatorial frameworks (e.g., the Chou–Talalay method), report complete concentration–response matrices, and measure mechanistic biomarkers. Although the modes of action of SF are manifold and diverse, the most consistent and robust mechanism identified is its upregulation of the Keap1/Nrf2/ARE pathway, which plays a crucial role in protection against cancer and numerous chronic or non-communicable diseases prevalent in contemporary Western society.

Furthermore, blood draws, the most common assessment method in allopathic medicine, allow for the straightforward evaluation of both basal and inducible Nrf2 activity in blood and other biofluids. Since there are literally hundreds of pathways upregulated by Nrf2, achieving consensus on the most effective biomarkers for comparative analyses between studies is essential. As highlighted in a recent review of Nrf2 scholarship “An ability to monitor basal and inducible Nrf2 signaling is vital for mechanistic understanding in translational studies” [224]. This review examined 1625 candidate genes and proteins, ultimately distilling them down to a core set of six markers. A follow-up 2025 report from the same team more deeply examines these 6 markers in a clinical context [17]. The 6-biomarker panel directly regulated by Nrf2 across multiple species includes glutamate-cysteine ligase catalytic subunit (GCLC), glutamate-cysteine ligase modifier subunit (GCLM), HO-1, NQO1, sulfiredoxin 1 (SRXN1), and thioredoxin reductase 1 (TXNRD1); all are critical components of the cellular antioxidant defense mechanism that hold varying significance for rapidly dividing cancer cells compared to normal cells.

### 9.4. Supplementation vs. Dietary Consumption

The epidemiologic literature on diet and cancer is now well seasoned, extensive and deep, and it highly implicates certain vegetables, in particular cruciferous vegetables and broccoli, in protection against cancer and a number of other chronic conditions [225,226]. It was in fact just this epidemiologic literature which led us to our decades-long investigation of SF [1,172,227,228,229,230]. The concept of “Food as Medicine” has unfortunately only started to gain traction in the medical education community very recently, and many nutrition-oriented scientists and physicians have been lamenting that fact for years [231,232]. At the time, we commented that in the early 2000s “preventive medicine specialists represented only 0.8 percent of the physician workforce and 0.5 percent of medical school faculty were trained in public health, preventive medicine, or related subspecialties” [232].

Supplementation, on the other hand, has also had a long history, but did not make it to mainstream medicine as soon as dietary interventions did. Since many or most dietary supplements (a.k.a. nutritional supplements) consisted of complex mixtures, extracts, emmolients, concoctions, and elixirs, they by definition contained many phytochemicals and, in many cases, other synthetic components, solvents, etc. They were also not well regulated, nor were they subject to much third party or even manufacturer testing. Scientists shied away from them since: (a) funding was extremely limited and obviously not supported by pharmaceutical companies, (b) the statistical and experimental ability to handle the effects of multiple bioactive compounds was limited, but is now becoming much less so, and (c) reputational damage had been done to supplements based on spurious and dangerous claims that were made prior to the enactment of the 1994 Dietary Supplement Health and Education Act. Beginning in the early 2000s the medical community began to appreciate the rigor and the demanding and lengthy scientific process that was required to establish the safety and effectiveness of developing medicinal agents and supplements from plants [233].

An unavoidable conclusion these days is that at least in the West, ultraprocessed “food” and the radical change in dietary habits over the past 2–5 decades have led to reduced fruit and vegetable intake, less variety, and hence reduced phytochemical intake over what our ancestors had consumed. This quite logically leads to the conclusion that supplementation is in order for some people (especially an older demographic), to provide phytonutrients, as well as vitamins and minerals, that may be missing from the diet. Although the need for supplementation of any sort remains hotly debated in scientific circles, supplementation is the fodder for an industry now estimated at in excess of USD 300 billion [234]. It is thus incumbent on responsible nutrition scientists to engage in the discussion or debate, and to evaluate the scientific evidence for specific supplements that has developed and will continue to develop at an even more rapid pace given the computational and analytic tools that can be brought to bear upon all related questions. It is also incumbent upon dieticians, nutritionists, policymakers, and the medical community to be prepared with well-informed answers to the lay public’s questions and concerns about supplementation.

## 10. Clinical and Therapeutic Implications

### 10.1. Oncology

SF’s ability to sensitize tumor cells to chemotherapy and protect normal tissues, particularly the heart, suggests two complementary clinical strategies:

1. **Dose-sparing enhancement:** Incorporate SF to reduce the required chemotherapy doses while maintaining efficacy and minimizing toxicity. For instance, lowering anthracycline doses could preserve tumor control while reducing the risk of cardiomyopathy. Additionally, some patients may be unable to receive the full dose of chemotherapy for reasons such as low white blood cell counts, either from the start of chemotherapy or as a result of cumulative side effects necessitating a “chemo break”. Thus, SF-driven dose-sparing could provide enhanced options for patients who have limited alternatives to chemotherapy.
2. **Overcoming resistance:** Utilize SF in refractory cases where epigenetic or redox mechanisms drive chemoresistance, employing biomarker-driven selection strategies. For example, tumors with epigenetic silencing may be amenable to HDAC/DNMT modulation. It is entirely reasonable to expect that a synergistic combination of SF administered alongside the initiation of chemotherapy could forestall and/or prolong time to the onset of drug resistance, thereby extending the time before disease progression. However, to our knowledge, this approach has not yet been tested in any clinical models.

Studies should not only assess response rates using the various metrics discussed herein but, if possible, also evaluate progression-free survival alongside toxicity reduction.

### 10.2. Non-Oncology Indications

Among the many possibilities, in the context of neuropathic pain and neuroinflammatory conditions, SF may be evaluated as an opioid-sparing adjuvant or as a disease-modifying agent via Nrf2/NLRP3 modulation. For metabolic diseases, SF could be explored as an adjunct to lifestyle interventions and pharmacotherapy aimed at improving insulin sensitivity and reducing hepatic gluconeogenesis.

## 11. Recommendations and Future Directions for Both Trials and Patients

Clinical trials should be randomized, include PK/PD endpoints, and pre-specify synergy endpoints.

1. **Standardize formulations.** Trials should utilize well-characterized SF or glucoraphanin/myrosinase products with documented bioavailability to ensure consistency.
2. **Biomarker-driven designs.** Incorporate PD markers, such as Nrf2 target induction, HDAC activity, and measurements of drug accumulation, metabolism, and excretion, into trial designs. Additionally, consider integrating metabolomics to better understand the impact of synergies.
3. **Rigorous synergy analysis.** Report comprehensive dose–response matrices and employ the Chou–Talalay [10] or Bliss independence [235] methods. Additionally, deposit raw data for independent re-analysis.
4. **Translational bridging studies.** Implement clinically relevant dosing in animal models and conduct early-phase human PK/PD studies prior to large-scale efficacy trials.
5. **Multi-arm pragmatic trials.** Consider using factorial designs to test the efficacy and toxicity reduction in SF combined with standard therapies simultaneously.
6. **“*n* of 1” and ecological studies.** These approaches hold significant potential for identifying prospective synergies.
7. **Tumor context stratification.** To propose SF synergies in clinical settings, oncologists can now utilize whole genome sequencing to assess tumor Nrf2 status and the epigenetic landscape, thereby identifying possible inadvertent tumor protection due to Nrf2 induction.

## 12. Conclusions

SF is a uniquely versatile phytochemical whose combined use with pharmaceuticals and other phytochemicals consistently yields synergistic outcomes across various cancer types and several non-cancer conditions. The mechanistic breadth of SF’s actions encompasses multiple pathways, many of which have not been directly tested with synergistic compounds, including Nrf2 activation, immune response modulation, antioxidant activity, regulation of cell cycle arrest, autophagy and apoptosis, inhibition of CYPs, selective antibiosis (against both bacteria and viruses), upregulation of the heat shock response, epigenetic modulation, and effects on drug transport. These mechanisms underpin the capacity of SF to potentiate anticancer efficacy while simultaneously protecting normal tissues from toxicity. Delving deeper, some of these effects involve the *inhibition* of NF-κB, HDACs, P-glycoprotein (Pgp), multidrug resistance-associated protein 1 (MRP-1), breast cancer resistance protein (BCRP), STAT3, mitogen-activated protein kinase kinase kinase 1 (MEKK1) activity, activator protein-1 (AP-1) DNA binding, and tubulin polymerization; the *degradation* of α- and β-tubulin; *downregulation* of cyclin B1, cyclin-dependent kinases (cdk1), cell cycle regulators (e.g., cdc25B, cdc25C), hypoxia-inducible factor (HIF), VEGF, and its receptor; *inhibition* of MMP-2 and MMP-9; *modulation* of Bcl-2 family proteins; and *activation* of caspases [4]. Translational progress will necessitate the standardization of GR or SF formulations, rigorous combination analyses, biomarker-driven trial designs, and attention to context-specific risks (e.g., potential tumor-protective effects of Nrf2 in certain cancers).

It should be noted that there is currently only a single clinical trial underway [21], with no results yet published, which aims to explore the synergistic actions of SF that are now so well outlined in vitro and in animal models. This trial is seeking to examine the protective effect of Avmacol, a GR plus myrosinase-based commercial supplement, on doxorubicin cardiotoxicity in 70 doxorubicin-naïve women with breast cancer undergoing neoadjuvant chemotherapy [80]. There is also a very small, non-registered human study that has been published, claiming enhanced antitumor response of CAR-T cells in patients with cancer who received adoptive immunotherapy [147]. With thoughtfully designed clinical trials, SF-containing strategies hold promise not only for the nutritional support of healthy individuals but also as integrated adjuncts to conventional therapies across oncology and other therapeutic areas.

Today’s computational and analytical tools, combined with artificial intelligence, now make it possible to conduct such research with a high probability of substantiating and supporting the already impressive in vitro and in vivo experimental literature.

## Figures and Tables

**Table 1:** Representative examples of sulforaphane synergy with pharmaceuticals in disease models along with key reference(s). * An “X” indicates that the Chou–Talalay Model was used by the study’s authors to quantify synergy. The Chou–Talalay combination index (CI; or CDI—combination drug index) theorem offers quantitative definition for additive effect (CI = 1), synergism (CI < 1), and antagonism (CI > 1) in combinations [9,10]. TNBC—Triple Negative Breast Cancer; SCC—squamous cell carcinoma.

Drug or Treatment Modality	Cancer Type or Condition	Reference	Chou–Talalay Metrics? *
CB-5083	acute myeloid leukemia	[120]	X
5-fluorouracil	adenoid cystic carcinoma	[121]	X
TRAIL-resistance	bladder	[86]	
paclitaxel	Breast (TNBC)	[75]	
docetaxel	Breast (TNBC)	[75]	
gemcitabine	breast	[122]	X
clofarabine	breast	[123]	
afimoxifene	breast	[124]	X
Lapatinib	breast	[125]	X
exemestane	breast	[126]	X
withaferin A	breast	[127]	X
genestein	breast	[116]	X
piperine	breast	[128]	
thymoquinone	breast	[128]	
biochanin A	breast	[129]	
genestein	breast	[117]	X
sodium butyrate	breast	[117]	X
doxorubicin	breast	[79]	
doxorubicin	breast	[100]	X
doxorubicin	breast (TNBC)	[108]	X
teriflunomide	breast (TNBC)	[105]	X
cisplatin	breast (TNBC)	[130]	X
5-fluorouracil	breast (TNBC)	[131]	X
morphine	cancer-induced bone pain	[132]	
hyperalgesic (antinociceptive)	cancer-induced bone pain	[132]	
17-beta estradiol	cardioprotection	[133]	
Doxorubicin	cardiac dysfunction	[80]	
eugenol	cervical	[134]	X
PNA-a15b	colon	[135]	X
curcumin	colon	[78]	X
dihydrocaffeic acid	colon	[78]	X
lycopene	colon	[136]	
quercetin	colon	[136]	
curcumin	colon	[136]	
selenium	colon	[137]	
selenium	colon	[138]	
oxaliplatin	colorectal	[139]	X
diindolylmethane (DIM)	colorectal	[140]	X
epigallocatechin-3-gallate (EGCG)	colorectal	[141]	X
dibenzoylmethane (DBM)	colorectal	[142]	
5-fluorouracil	colorectal	[131]	X
cisplatin	colorectal	[143]	
salinomycin	colorectal	[92]	X
torkinib (PP242)	esophageal SCC	[30,144]	X
temozolomide	glioblastoma	[30]	
resveratrol	glioma	[145]	
temozolomide	glioblastoma	[30]	X
PNA-a15b	glioma/glioblastoma	[146]	X
CART-T cell therapy	hematologic malignancies	[147]	X
auranofin	hepatocellular	[148]	
gemcitabine	intrahepatic cholangiocarcinoma	[149]	X
imatinib	leukemia	[150]	
allyl isothiocyanate	lung	[151]	X
gefitinib	lung	[152]	
gefitinib	lung	[153]	X
cisplatin	mesothelioma (malignant)	[154]	
arsenic trioxide	multiple myeloma	[91]	X
myrecetin	obesity (adipocytes)	[155]	
cisplatin	pancreatic	[156,157,158]	X
gemcitabine	pancreatic	[156]	
doxorubicin	pancreatic	[156,157,158]	X
5-fluorouracil	pancreatic	[156,157,158]	X
17-allylamino 17-demethoxygeldanamycin	pancreatic	[157]	X
ibuprofen	pancreatic	[157,159]	X
aspirin	pancreatic	[98]	
curcumin	pancreatic	[98]	
sorafenib	pancreatic	[160]	
quercetin	pancreatic	[161]	
catechins	pancreatic	[161]	
loratadine	pancreatic	[162]	
taxol (paclitaxel)	prostate	[156]	
cisplatin	prostate	[156]	
bicalutamide	prostate	[163,164]	X
enzalutamide	prostate	[163,164]	X
TRAIL	prostate	[88]	
ganetespib	prostate	[164]	X
TRAIL	rhabdosarcoma	[84]	
cisplatin	skin (epidermal SCC)	[165]	
Fernblock XP (fern extract)	skin (melanoma and aging)	[166]	X
quercetin	skin (melanoma)	[167]	
luteolin	general inflammation	[168]	X

## Data Availability

No new data were created or analyzed in this study. Data sharing is not applicable to this article.

## Supplementary Materials

The following supporting information can be downloaded at: https://www.mdpi.com/article/10.3390/medicines13020016/s1. Supplementary Table S1. Clinical studies in which glucoraphanin, sulforaphane, or some form of broccoli or broccoli sprout extract was used in one of the intervention arms. Arranged in order of NCT registry number (clinicaltrials.gov) if registered, or author first name if not registered. A key to abbreviations and summary metrics follows the table, which was updated as of 15 March 2026. A spreadsheet version is available upon request from jfahey@jhmi.edu or hliu8@jhmi.edu. References [13,14,15,16,48,189,190,191,203,236,237,238,239,240,241,242,243,244,245,246,247,248,249,250,251,252,253,254,255,256,257,258,259,260,261,262,263,264,265,266,267,268,269,270,271,272,273,274,275,276,277,278,279,280,281,282,283,284,285,286,287,288,289,290,291,292,293,294,295,296,297,298,299,300,301,302,303,304,305,306,307,308,309,310,311,312,313,314,315,316,317] are cited in the Supplementary Materials.

## Abbreviations

The following abbreviations are used in this manuscript:

Akt	protein kinase B
ALDH	aldehyde dehydrogenase
AP-1	activator protein-1
BAX	Bcl-2 Associated X protein
Bcl-2	B-cell lymphoma 2
BCRP	breast cancer resistance protein
BSE	broccoli sprout (or seed) extract
CAR-T	chimeric antigen receptor cell therapy
CB-5083	p97 inhibitor drug
CD {CD34, 38, 44, or 133}	cluster of differentiation
cdc25 {B and C}	cell division cycle 25 phosphatases
CDK	cyclin-dependent kinase
CHOP	DNA damage-inducible transcript 3
CI	combination index
CIBP	cancer-induced bone pain
COX-2	cyclooxygenase-2
CSC	cancer stem cell
DBM	dibenzoyl methane
DIM	3,3′-diindolylmethane
DNMT	DNA methyltransferase
DOX	doxorubicin
EGCG	epigallocatechin gallate
EMT	epithelial-to-mesenchymal transition
ERK	extracellular signal-regulated kinase
FAP	familial adenomatous polyposis
GE	genistein
GEM	gemcitabine
GPx2	glutathione peroxidase 2
GR	glucoraphanin
GSK3β	glycogen synthase kinase-3 beta
HDAC	histone deacetylase
HER2	human epidermal growth factor receptor-2
HIF	hypoxia-inducible factor
HO-1	heme oxygenase 1; HMOX-1
Hsp 90	heat shock protein 90
hTERT	human telomere reverse transcriptase
iCCA	intrahepatic cholangiocarcinoma
IL-1β	interleukin-1 beta
IL-6	interleukin-6
iNOS	inducible nitric oxide synthase
Keap1	Kelch-like ECH-associated protein 1
Ki-67	marker of proliferation Kiel 67
LC3B	microtubule-associated protein 1 light chain 3β
MAPK	mitogen-activated protein kinase
MEKK1	mitogen-activated protein kinase kinase kinase 1
miR {-30a and -124}	small non-coding RNAs
MMP	matrix metalloproteinases
MOR	mu opioid receptor
mTOR	mechanistic target of rapamycin
NaB	sodium butyrate
Nanog	a homeobox protein transcription factor
NLRP3	nucleotide-binding domain, leucine-rich-containing family, pyrin domain-containing-3
Nrf2	NF-E2-related factor 2
NF-кB	nuclear factor-kappa B
NQO1	NAD(P)H quinone oxidoreductase 1
NTN4	netrin 4
Oct-4	octamer—binding transcription factor 4
p53	tumor protein p53
p57	cyclin-dependent kinase (CDK) inhibitor
p97	valosin-containing protein
PARP	poly(ADP-ribose) polymerase
PD	pharmacodynamic
Pgp	P-glycoprotein
PI3K	phosphatidylinositol 3-kinase
PK	pharmacokinetic
PNA-a15b	a peptide nucleic acid designed to target and inhibit miR-15b-5p
PUMA	p53-up-regulated modulator of apoptosis
RB	retinoblastoma protein
ROS	reactive oxygen species
SALL1	Sal-like 1; spalt-like transcription factor 1
SF	sulforaphane
SHH	sonic Hedgehog
SIRT	sirtuin
SOD	superoxide dismutase
SOX2	sex determining region Y-box2
STAT3	signal transducer and activator of transcription 3
TrxR1	thioredoxin reductase 1
TNBC	triple-negative breast cancer
TNF-α	tumor necrosis factor-alfa
TRAIL	tumor necrosis factor-related apoptosis-inducing ligand (Apo2L)
VEGF	vascular endothelial growth factor
Wnt	a family of glycoproteins
ZO-1	zonula occludens-1
