# Supplementary material for: Sulforaphane Synergies with Phytochemicals and Pharmaceuticals: Implications for Healthspan
Source: Medicines (Basel). 2026 May 6;13(2):16. doi: 10.3390/medicines13020016 (PMC13214911; doi:10.3390/medicines13020016)
Supplement: Supplementary file 1 [file medicines-13-00016-s001.zip › medicines-4168785-supplementary.pdf]

Supplementary Data for:

Review

Sulforaphane Synergies with Phytochemicals and Pharmaceuticals: Implications for Healthspan

Jed W. Fahey [1,2,3,4,5]* and Hua Liu [6]

[1] Department of Medicine, The Johns Hopkins University School of Medicine, Baltimore, MD 21205, USA
[2] Department of Psychiatry & Behavioral Sciences, The Johns Hopkins University School of Medicine, Baltimore, MD 21205, USA
[3] Department of Physiology, Pharmacology & Therapeutics, The Johns Hopkins University School of Medicine, Baltimore, MD 21205, USA
[4] iMIND Institute, The Johns Hopkins University School of Medicine, Baltimore, MD 21205, USA
[5] Institute of Medicine, University of Maine, Orono, ME 04469, USA
[6] Stanley Division of Developmental Neurovirology, Department of Pediatrics, The Johns Hopkins University School of Medicine, Baltimore, MD 21287, USA
* Correspondence: jfahey@jhmi.edu

Supplementary Data

Supplementary Table S1. Clinical studies in which glucoraphanin, sulforaphane, or some form of broccoli or broccoli sprout extract was used in one of the intervention arms. Arranged in order of NCT registry number (clinicaltrials.gov) if registered, or author first name if not registered. A key to abbreviations and summary metrics follows the table, which was updated as of March 15, 2026. A spreadsheet version is available upon request from jfahey@jhmi.edu or hliu8@jhmi.edu .

No.	Year Published or Last Updated at Clinicaltrials.gov	First Author (If published), or P.I., or Sponsor	Study Title or Study Population	Treatment	Indication/Condition/Target Population	NCT Number if Registered	Ref.
1	2026	Imdea Food	Plant miRNAs In Human Plasma After Broccoli Consumption	unspecified supplement	gut microbiota miRNAs	NCT07456267	
2	2026	Lithuanian Sports University	Short-Term Broccoli Supplementation and Acute Oxidative Stress Recovery	BrocAffex	athletes	NCT07454265	
3	2026	Lithuanian Sports University	Short-term Effects of Broccoli-derived Glucoraphanin on Recovery From Eccentric Muscle Damage	unspecified	non-athletes	NCT07373106	
4	2026	Mazzo, E (IRCCS)	Myrosinase Bioactivated Gglucoraphanin for the Treatment of Neurodegenerative Diseases (GRA-MYR-ND)	Nomio	MS, PD	NCT07360977	
5	2023	University of Maia	Effects of Sulforaphane Supplementation on Athletic Performance	Nomio	trained athletes	NCT07343518	
6	2026	Lee, SY (Pusan University)	Broccoli Sprout Extract for Cognitive Function	Unspecified BSE supplement	cognitive function	NCT07334366	

7	2025	Sun, Zimin	Safety and Feasibility of Sulforaphane to Promote Early Haematopoietic Recovery After Cord Blood Transplantation	Unspecified Supplement	at risk for hematological malignancies	NCT07297576	
8	2025	Ou, J-J	Mechanism Study of Sulforaphane in ASD Improvement	Avmacol	ASD	NCT07047976	
9	2025	Kirkwood (UPMC)	Testing the Effect of Sulforaphane, a Compound Naturally Found in Cruciferous Vegetables, on Preventing Melanoma in Patients With a Prior History of Melanoma	Avmacol ES	at risk for melanoma	NCT07040280	
10	2025	Li, Yuanyuan (U of MD)	Metabotyping of Broccoli Microgreen in Obese Breast Cancer Survivors	BS	obesity	NCT07032545	
11	2026	Papillex	A Clinical Trial to Investigate the Safety and Efficacy of PapillexÂ® on Abnormal Cervical Cells Caused by HPV.	multi-supplement w/ BS powder	at risk for cervical cancer	NCT06979180	
12	2025	M Jesus Periago	Chronic Intervention With Sulforaphane-SmartÂ® in Overweight and Obese Adults	SF-Smart (Ingredala)	overweight and obese	NCT06964659	
13	2025	M Jesus Periago	Acute Intervention with Sulforaphane-SmartÂ®	SF-Smart (Ingredala)	PK	NCT06856486	
14	2026	Roswell Park Cancer Inst.	A Cruciferous Vegetable Eating Program for the Reduction of Cancer Recurrence and Progression in Patients With Non-muscle Invasive Bladder Cancer	broccoli	Bladder Cancer	NCT06733363	
15	2024	Assistance Publique - Hopitaux de Paris	Study of the Efficacy of Sulforaphane in Children Aged 6 to 12 With Attention Deficit Disorder With or Without Hyperactivity	Sulfodyne BSdE	ADHD	NCT06594536	
16	2025	Torquati, L (U of Exeter)	Effect of L. Plantarum Probiotic Supplementation on Broccoli Sulforaphane Bioavailability: Randomised Double-blind	Source Naturals BSE + probiotic	microbiome	NCT06561893	

			Placebo-controlled Crossover Study				
17	2023	Standard Process, Inc.	Tolerance and Palatability of 10-day Supplementation With Hemp Oil, Calamari Oil, and Broccoli Extract in Healthy Adults	Hemp Oil w/ GR	Healthy	NCT06081140	
18	2026	Savani, M	Evaluation of Broccoli Seed and Sprout Extract for Detoxification of Carcinogens in Firefighters	Avmacol ES	At-risk of carcinogenesis	NCT06009926	
19	2023	Zaparte, Aline	Broccoli Extract Supplementation in Older Adults With Alcohol Use Disorder	Avmacol	alcohol use disorder	NCT05902754	
20	2023	Le, Thu (UofRochester)	Safety, Feasibility and Efficacy of Sulforaphane (Avmacol Extra Strength) in Chronic Kidney Disease	Avmacol ES	CK disease	NCT05797506	
21	2023	Olson, Camilla	Broccoli Seed Extract and Skin Health	TrueBroc	healthy	NCT05745636	
22	2024	Levy, Rebecca	Safety, Feasibility, and Tolerability of Sulforaphane in Children With Chronic Kidney Disease	unspecified supplement	children w/ CK disease	NCT05653492	
23	2026	Chen, Grace	Broccoli Sprouts for Mild Ulcerative Colitis	BS	ulcerative colitis	NCT05507931	
24	2022	Singh, Sharda	Prevention of Age-associated Cardiac and Vascular Dysfunction Using Avmacol ES	Avmacol	CVD	NCT05408559	
25	2022	Kroon, Paul	Broccoli Effect on Glycated Haemoglobin (HbA1c)	broccoli	pre-diabetes	NCT05367024	
26	2023	UC Davis	Open-Label Trial of Sulforaphane in Premutation Carriers With FXTAS	Avmacol	FXTAS	NCT05233579	
27	2024	Giron	Sulforaphane Use to Effect Inflammatory and Metabolic	TrueBroc	HIV	NCT05224492	[1]

			Changes in Virally Suppressed HIV Patients				
28	2024	Le, Thu	Study of Sulphoraphane in Chronic Kidney Disease	Avmacol ES	CK disease	NCT05153174	
29	2021	Liu, Xiaohua	A Pilot Study on Effect of add-on Sulforaphane to SSRIs and Application of Niacin Skin Flush Response Test in Major Depressive Disorder	Zhiyinguosu	depression	NCT05148169	
30	2022	Maastricht Univ.	The PROtective Effect of SulforAphaNe on Chronic Low-grade Inflammation in Healthy Participants	BroccoCress	inflammation	NCT05146804	
31	2021	Liu, Xiaohua	A Comparative Study on Efficacy and Safety of add-on Sulforaphane or rTMS to Escitalopram for Major Depressive Disorder With Poor Response to Initial Treatment	Zhiyinguosu	depression	NCT05145270	
32	2021	Bauman, Julie	Testing the Effect of the Broccoli Seed and Sprout Extract, Avmacol ES, on the Cancer Causing Substances of Tobacco in Heavy Smokers	Avmacol	lung cancer	NCT05121051	
33	2023	Wu, Renrong	A 6-month Study to Evaluate Sulforaphane Effects in PD Patients	Avmacol	PD	NCT05084365	
34	2026	Mastaloudis	Exogenous myrosinase from mustard seed increases bioavailability of sulforaphane from a glucoraphanin-rich broccoli seed extract in a randomized clinical study	TrueBroc	bioavailability	NCT04946526	[2]
35	2025	Stenvinkel, Peter	Effect of Broccoli Sprout Extract in Patients With Chronic Kidney Disease With Diabetes Type 2	BSE	CK disease	NCT04858854	
36	2025	Traustadóttir, Tinna	Treatment Strategy to Enhance Nrf2 Signaling in Older Adults	EnduraCell Bioactive	aging	NCT04848792	

37	2025	Torres, E (Rutgers)	Validation Digital Bio-markers as a Tool to Measure Improvement in Core Symptoms of Autism During Sulforaphane Treatment.	Avmacol	ASD	NCT04805957	
38	2025	Ho, Emily	Discovery of Biological Signatures for Cruciferous Vegetable Intake (Single Serving)	broccoli	biomarkers	NCT04641026	
39	2025	Univ. Federal Fluminense	Effects of Sulforaphane for Patients With Chronic Kidney Disease	"Dietary Supplement: L-sulforaphane 1%"	CK disease	NCT04608903	
40	2022	Hei, 2022; Huang, 2025	A 6-month Study to Evaluate Sulforaphane Effects in Schizophrenia Patients	Avmacol	SZ	NCT04521868	[3, 4]
41	2022	Univ. of Oxford	Sulforaphane Supplementation Study (FAMOUS)	BroccoMax	NAFLD	NCT04364360	
42	2020	Xiangya Hospital	Effects of Sulforaphane on Cognitive Function in Patients With Frontal Brain Damage	Avmacol	cognition	NCT04252261	
43	2020	Zhejiang Univ.	Effects of Sulforaphane in Patients With Prodromal to Mild Alzheimer's Disease	Wulab-AD sulforaphane	AD	NCT04213391	
44	2024	Texas Tech Univ.	Doxorubicin-naïve women with breast cancer	Avmacol	doxorubicn-associated cardiomyopathy	NCT03934905	
45	2021	Li	Adults at risk for psychosis	Zhiyinguosu	psychosis	NCT03932136	[5]
46	2017	Davidson	osteoarthritis	broccoli	osteoarthritis	NCT03878368	[6]
47	2019	Kaczmarek	Bioavailability of Phytonutrients From Novel Preparations of Broccoli	broccoli	bioavailability	NCT03773497	[7]
48	2023	Bauman	Smokers	Avmacol	lung cancer	NCT03402230	[8]
49	2025	Chien	healthy adults	Crucera-SGS	skin inflammation	NCT03289832	[9]
50	2020	Zandberg	Head & neck cancer patients post-curative treatment	Avmacol	head and neck cancer	NCT03268993	

51	2025	Yuan	Former Smokers	Avmacol	lung cancer	NCT03232138	[10]
52	2020	Chang	*Helicobacterpylori* infected adults (18-75 y.o.)	BSE	H. pylor (stomach cancer)	NCT03220542	[11]
53	2021	Bauman	Head and Neck Cancer Survivors	Avmacol	head and neck SCC	NCT03182959	
54	2020	Politte. Laura	Young men (13-30 y.o.), on the autism spectrum	Avmacol	ASD	NCT02909959	
55	2021	Yusin	Veterans with allergic rhinitis	BSE	allergic rhinitis	NCT02885025	[12]
56	2022	Hei	Adults with 1st episode or early onset schizophrenia (SZ)	Avmacol	SZ	NCT02880462	[3]
57	2020	Ou	Children (3-15 y.o.) on the autism spectrum	Avmacol	ASD	NCT02879110	[13]
58	2020	Dickerson	Adults (18-65 y.o.) with szhizophrenia	Avmacol	SZ	NCT02810964	[14, 15]
59	2021	Geiger	Bioavailability and Mucosal Bioactivity of AvmacolÂ® in Healthy Volunteers	Avmacol	bioavailability	NCT02800265	[16]
60	2025	Buyske, Steven	Young adults (13-30 y.o.), on the autism spectrum	Avmacol	ASD	NCT02677051	
61	2020	Zimmerman	Children (3-12 y.o.), on the autism spectrum	Avmacol	ASD	NCT02561481	[17]
62	2018	Sivapalan	The Bioavailability Of Sulforaphane From Broccoli Soups Study (BOBS)	broccoli	bioavailability	NCT02300324	[18]
63	2016	Bauman	Prevention of Carcinogen-Induced Oral Cancer by Sulforaphane	BSE-SF	oral cancer	NCT02023961	[19]
64	2012	Universidad Politécnica de Cartagena	Bioavailability of Chemopreventive and Nutritional Compounds in Broccoli	broccoli	bioavailability	NCT01743924	
65	2016	Doss	sickle cell disease (SCD)	BS	sickle cell disease	NCT01715480	[20]
66	2019	Chartoumpekis	healthy women	BS	safety (healthy adults)	NCT01437501	[21]
67	2010	Brahmer, J	Broccoli Sprout Extract in Preventing Lung Cancer in Smokers	BSE-SF	lung cancer	NCT00255775	

68	2025	Vinge	Healthy	GR	exercise		[22]
69	2023	Flockhart, M	Healthy	BS	exercise		[23]
70	2019	Lopez-Chillon	overweight	BS	overweight and obese		[24]
71	2019	Chen	Healthy	BS	air pollutant detoxication		[25]
72	2019	Fahey	Healthy	Avmacol	bioavailabiliy		[26]
73	2019	Traka	men with low to intermediate risk of prostate cancer	broccoli	prostate cancer		[27]
74	2018	Sedlak	healthy	BSE-SF	biomarkers		[28]
75	2018	Tahata	melanoma	BSE	melanoma		[29]
76	2018	Bent	Children with ASD and related neurodevelopmental disorders	Avmacol	ASD		[30]
77	2018	Housley	Healthy	BS	metabolomics/bioavailability		[31]
78	2018	Okunade	Healthy	broccoli	bioavailability		[32]
79	2017	Axelsson	diabetics (well regulated and dysregulated)	BSE-SF	T2D		[33]
80	2017	Fahey	healthy	SF-cyclodextrin; Prostaphane	bioavailability		[34]
81	2016	Duran	Healthy	BS	inflammation		[35]
82	2016	Müller	Healthy	BS	antiviral		[36]
83	2016	Sudini	asthma	BS	asthma		[37]
84	2016	Wise	COPD	BSE-SF	COPD		[38]
85	2015	Alumkal	prostate cancer	BSE-SF	prostate cancer		[39]
86	2015	Armah	CVD disease risk	broccoli	CVD		[40]
87	2015	Atwell	abnormal mammograms; scheduled for breast biopsy	BSdE (BroccoMax)	breast cancer		[41]
88	2015	Atwell	Healthy	BS	bioavailability		[42]
89	2015	Brown	moderate asthma	BSE-SF	asthma		[43]
90	2015	Chang	*Helicobacter pylori*	BSE	stomach cancer		[44]
91	2015	Cipolla	prostate cancer patients post-radical prostatectomy	prostaphane	prostate cancer		[45]

92	2015	Fahey	healthy	OncoPlex	bioavailability		[46]
93	2015	Kikuchi	elevated liver function markers with diagnosis of fatty liver	BS	NAFLD		[47]
94	2015	Medina	healthy	BS	inflammation and oxidative stress		[48]
95	2015	Shiina	schizophrenia	BSE	SZ		[49]
96	2015	Ushida	Healthy	BS	cancer prevention		[50]
97	2014	Bahadoran	Type 2 diabetic with H. pylori infection	BSP (Cyvex)	T2D		[51]
98	2014	Baier	Healthy	BS	depression		[52]
99	2014	Egner	Healthy	BSE-GR & BSE-SF	air pollution detoxification		[53]
100	2014	Heber	Healthy	BSE-SF	air pollution detoxification		[54]
101	2014	Noah	Healthy	BS	antiviral; air pollution; immune response		[55]
102	2014	Singh	aged 13–27 y; w/ moderate to severe Autism Spectrum Disorder	BSE-SF	ASD		[56, 57]
103	2013	Armah	have a 10-y CVD risk profile	broccoli	CVD		[58]
104	2013	Meyer	Healthy	BSE-SF	immune response; air pollution		[59]
105	2013	Poulton	Healthy	BSE-SF	PK/PD		[60]
106	2012	Bahadoran	Type 2 diabetes	BroccoPhane	T2D		[61]
107	2012	Cramer	Healthy	BS & GR	bioavailability		[62]
108	2012	Fahey	healthy	BSE-GR	bioavailability		[63]
109	2012	Kensler	Healthy	BSE-GR & BSE-SF	air pollution detoxification		[64]
110	2012	Mirmiran	Type 2 diabetes	BroccoPhane	T2D		[65]
111	2012	Saha	Healthy	broccoli	bioavailability		[66]
112	2011	Bahadoran		BroccoPhane	T2D		[67]
113	2011	Clarke	healthy non-smokers	BS or ?	PK/PD		[68]

114	2011	Egner	Healthy	BSE-SF	bioavailability		[69]
115	2010	Christiansen	hypertensive, w/o diabetes & w/ normal cholesterol	BS	CVD		[70]
116	2009	Hanlon	Healthy	broccoli	PK		[71]
117	2009	Riedl	Healthy	BSE-SF	PK/PD		[72]
118	2009	Riso	Healthy (10 smokers and 10 nonsmokers)	broccoli	lung cancer		[73]
119	2009	Yanaka	H. pylori–infected	BSE-GR	stomach cancer		[74]
120	2008	Traka	Diagnosed with high-grade prostatic intraepithelial neoplasia (PIN)	broccoli	prostate cancer; PK/PD		[75]
121	2008	Vermeulen	Healthy	broccoli	bioavailability; PK		[76]
122	2007	Talalay	healthy	BSE-SF	skin inflammation		[77]
123	2007	Cornblatt	reduction mammaplasty	BSE-SF	breast cancer		[78]
124	2007	Gasper	Healthy	broccoli	stomach cancer; PK/PD		[79]
125	2007	Myzak	Healthy	BS	cancer prevention		[80]
126	2007	Rungapamestry	Healthy	broccoli	bioavailability		[81]
127	2006	Shapiro	Healthy	BSE-GR & BSE-SF	safety PK/PD		[82]
128	2005	Gasper	Healthy	broccoli	bioavailability; PK/PD		[83]
129	2005	Kensler	Healthy	BSE-GR	liver cancer; PK/PD		[84]
130	2004	Murashima	healthy	broccoli	pharmacodynamics; oxidative stress		[85]
131	2004	Walters	Healthy	broccoli	pharmacodynamics		[86]
132	2004	Galan	*Helicobacter pylori* infected adults	BS	stomach cancer		[87]
133	2002	Ye	healthy	BSE-SF	PK		[88]
134	2001	Hauder	Healthy male non-smoking, aged 50-82 y)	broccoli	PK		[89]

135	2000	Conaway	Healthy	broccoli	PK; bioavailability		[90]
136	1998	Shapiro	Healthy	broccoli	safety; PK		[91]

<u>Total # of Studies - 136</u>

Avmacol (reg. or ES) - 27
Broccoli - 24
BrocAffex -.. 1
BroccoCress -.................................. 1
BroccoMax - 2
BroccoPhane -.................................. 3
BS (broccoli sprouts) - 25
BSE (broccoli sprout extract) -.......... 7
Crucera-SGS.................................... 1
Cyvex - 1
EnduraCell Bioactive -...................... 1
BSE-GR (JHU GR-rich BSE) - 7
BSE-SF (JHU SF-rich BSE) - 18
GR –.. 2
Nomio -.. 2
Oncoplex - 1
Prostaphane -.................................. 2
SF-cyclodextrin - 1
SF-Smart -.. 2
Source Naturals - 1
Sulfodyne - 1
TrueBroc - 3
Wulab-AD sulforaphane - 1
Zhiyinguosu -.................................... 3
Unspecified or other - 5

Treatment / Intervention

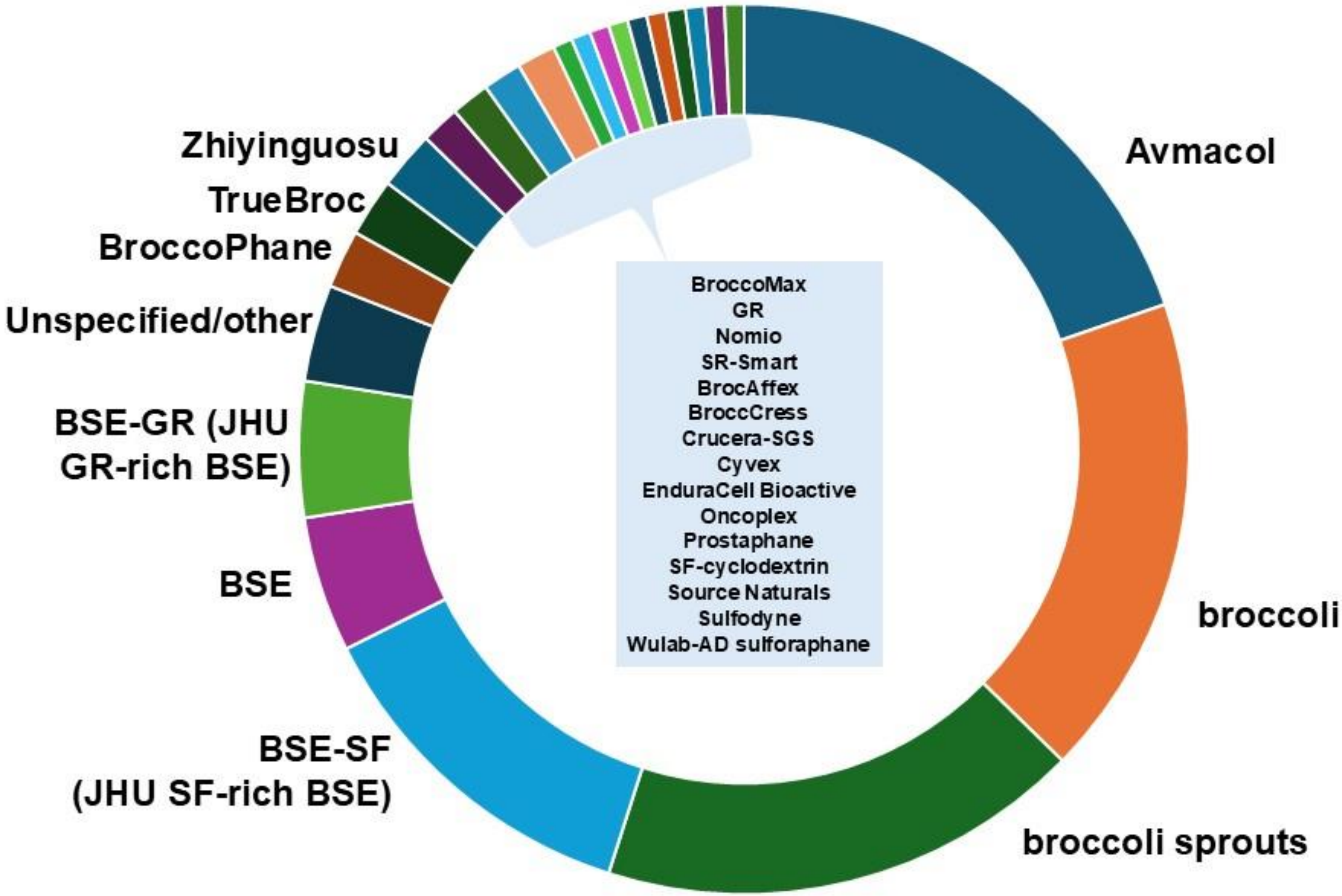

Indication

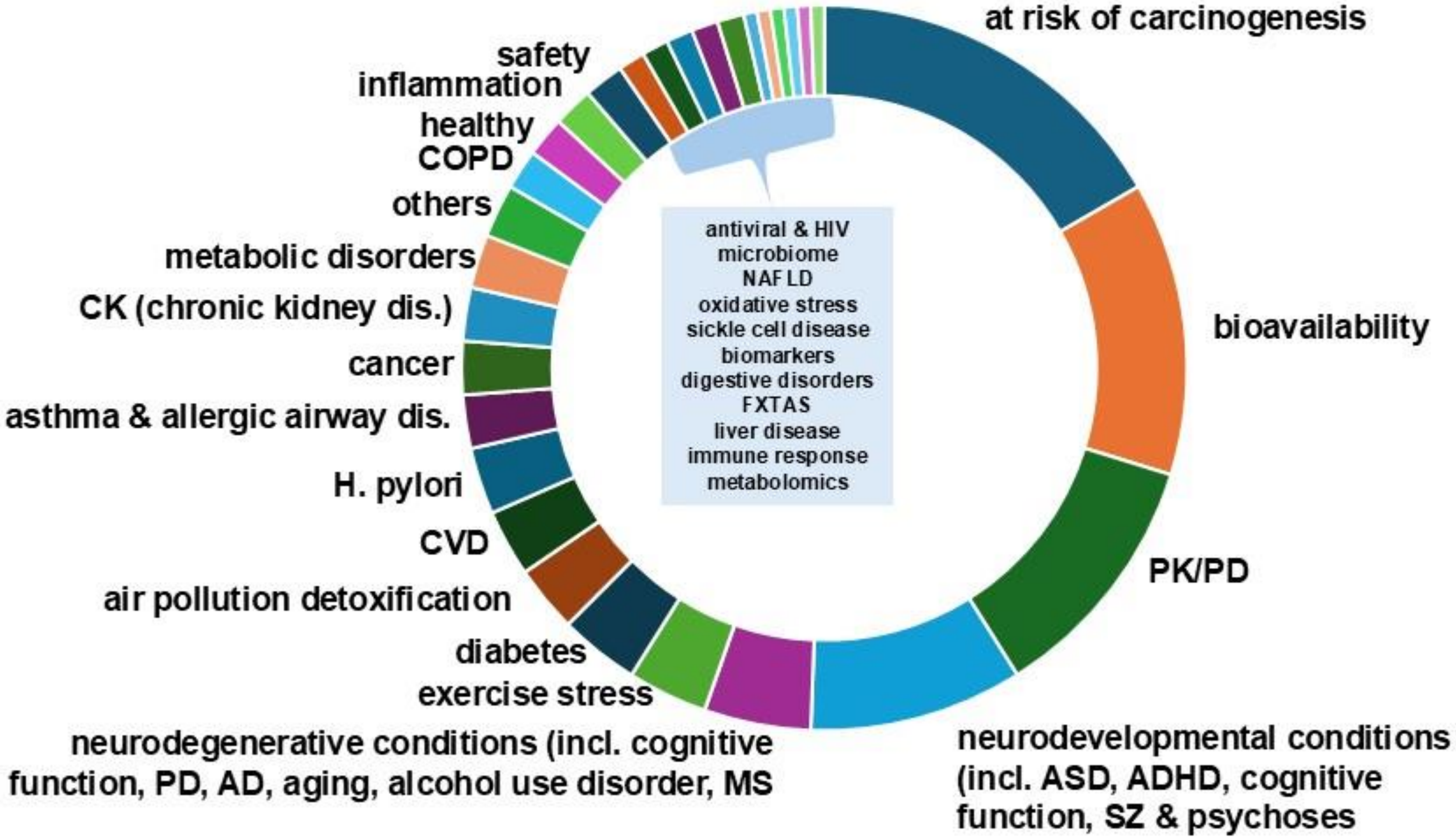

at risk of carcinogenesis
bioavailability
PK/PD
neurodevelopmental conditions (incl. ASD, ADHD, cognitive function, SZ & psychoses
neurodegenerative conditions (incl. cognitive function, PD, AD, aging, alcohol use disorder, MS
exercise stress
diabetes
air pollution detoxification
CVD
H. pylori
asthma & allergic airway dis.
cancer
CK (chronic kidney dis.)
metabolic disorders
others
COPD
healthy
inflammation
safety
antiviral & HIV
microbiome
NAFLD
oxidative stress
sickle cell disease
biomarkers
digestive disorders
FXTAS
liver disease
immune response
metabolomics

Reference List for Supplementary Table S1.

1. Giron, J.; Smiarowski, L.; Katz, J., The effect of sulforaphane on markers of inflammation and metabolism in virally suppressed HIV patients. *Front Nutr* **2024,** *11*, 1357906.
2. Mastaloudis, A.; Holcomb, L.; Fahey, J. W.; Olson, C.; Nieman, D. C.; Kay, C.; O'Donnell, R.; Pecorelli, A.; Kinney, M.; Li, Y.; Ishaq, S. L.; Valacchi, G., Exogenous myrosinase from mustard seed increases bioavailability of sulforaphane from a glucoraphanin-rich broccoli seed extract in a randomized clinical study. *Sci. Rep.* **2026**.
3. Hei, G.; Smith, R. C.; Li, R.; Ou, J.; Song, X.; Zheng, Y.; He, Y.; Arriaza, J.; Fahey, J. W.; Cornblatt, B.; Kang, D.; Yang, Y.; Huang, J.; Wang, X.; Cadenhead, K.; Zhang, M.; Davis, J. M.; Zhao, J.; Jin, H.; Wu, R., Sulforaphane Effects on Cognition and Symptoms in First and Early Episode Schizophrenia: A Randomized Double-Blind Trial. *Schizophr Bull Open* **2022,** *3* (1), sgac024.
4. Huang, J.; Chen, A.; Jin, H.; Liu, F.; Hei, G.; Teng, Z.; Xiao, J.; Wu, R.; Zhao, J.; Davis, J. M.; Shao, P.; Smith, R. C., Efficacy and Safety of Sulforaphane Added to Antipsychotics for the Treatment of Negative Symptoms of Schizophrenia: A Randomized Controlled Trial. *J Clin Psychiatry* **2025,** *86* (1).
5. Li, Z.; Zhang, T.; Xu, L.; Wei, Y.; Tang, Y.; Hu, Q.; Liu, X.; Li, X.; Davis, J.; Smith, R.; Jin, H.; Wang, J., Decreasing risk of psychosis by sulforaphane study protocol for a randomized, double-blind, placebo-controlled, clinical multi-centre trial. *Early Interv Psychiatry* **2021,** *15* (3), 585-594.
6. Davidson, R.; Gardner, S.; Jupp, O.; Bullough, A.; Butters, S.; Watts, L.; Donell, S.; Traka, M.; Saha, S.; Mithen, R.; Peffers, M.; Clegg, P.; Bao, Y.; Cassidy, A.; Clark, I., Isothiocyanates are detected in human synovial fluid following broccoli consumption and can affect the tissues of the knee joint. *Sci Rep* **2017,** *7* (1), 3398.
7. Kaczmarek, J. L.; Liu, X.; Charron, C. S.; Novotny, J. A.; Jeffery, E. H.; Seifried, H. E.; Ross, S. A.; Miller, M. J.; Swanson, K. S.; Holscher, H. D., Broccoli consumption affects the human gastrointestinal microbiota. *J Nutr Biochem* **2019,** *63*, 27-34.
8. Bauman, J. E.; Hsu, C. H.; Centuori, S.; Guillen-Rodriguez, J.; Garland, L. L.; Ho, E.; Padi, M.; Bageerathan, V.; Bengtson, L.; Wojtowicz, M.; Szabo, E.; Sherry Chow, H. H., Randomized Crossover Trial Evaluating Detoxification of Tobacco Carcinogens by Broccoli Seed and Sprout Extract in Current Smokers. *Cancers* **2022,** *14* (9).
9. Chien, A. L.; Liu, H.; Rachidi, S.; Feig, J. L.; Wang, R.; Wade, K. L.; Stephenson, K. K.; Kecici, A. S.; Fahey, J. W.; Kang, S., Oral Glucoraphanin and Curcumin Supplements Modulate Key Cytoprotective Enzymes in the Skin of Healthy Human Subjects: A Randomized Trial. *Metabolites* **2025,** *15* (6).
10. Yuan, J. M.; Kensler, T. W.; Dacic, S.; Hartman, D. J.; Wang, R.; Balogh, P. A.; Sufka, P.; Turner, M. A.; Fuhrer, K.; Seigh, L.; Pham, Y. T.; Adams-Haduch, J.; Valacchi, G.; Singh, S. V.; Herman, J. G.; Wilson, D. O.,

Randomized Phase II Clinical Trial of Sulforaphane in Former Smokers at High Risk for Lung Cancer. *Cancer Prev Res (Phila)* **2025,** *18* (6), 335-345.
11. Chang, Y. W.; Park, Y. M.; Oh, C. H.; Oh, S. J.; Cho, J. H.; Kim, J. W.; Jang, J. Y., Effects of probiotics or broccoli supplementation on Helicobacter pylori eradication with standard clarithromycin-based triple therapy. *Korean J Intern Med* **2020,** *35* (3), 574-581.
12. Yusin, J.; Wang, V.; Henning, S. M.; Yang, J.; Tseng, C.-H.; Thames, G.; Arnold, I.; Heber, D.; Lee, R.-P.; Sanavio, L.; Pan, Y.; Qin, T.; Li, Z., The Effect of Broccoli Sprout Extract on Seasonal Grass Pollen-Induced Allergic Rhinitis. *Nutrients* **2021,** *13* (4), 1337.
13. Ou, J.; Smith, R. C.; Tobe, R. H.; Lin, J.; Arriaza, J.; Fahey, J. W.; Liu, R.; Zeng, Y.; Liu, Y.; Huang, L.; Shen, Y.; Li, Y.; Cheng, D.; Cornblatt, B.; Davis, J. M.; Zhao, J.; Wu, R.; Jin, H., Efficacy of Sulforaphane in Treatment of Children with Autism Spectrum Disorder: A Randomized Double-Blind Placebo-Controlled Multi-center Trial. *J Autism Dev Disord* **2024,** *54* (2), 628-641.
14. Dickerson, F.; Origoni, A.; Katsafanas, E.; Squire, A.; Newman, T.; Fahey, J.; Xiao, J. C.; Stallings, C.; Goga, J.; Khushalani, S.; Yolken, R., Randomized controlled trial of an adjunctive sulforaphane nutraceutical in schizophrenia. *Schizophr Res* **2021,** *231*, 142-144.
15. Dickerson, F., Efficacy of Add-On Sulforaphane for Improving Symptoms and Cognition in Schizophrenia: A Randomized Double-Blind Study. In *Schizophrenia International Research Society, SIRS*, 2021.
16. Geiger, J. L.; Cedars, E. D.; Zang, Y.; Normolle, D. P.; Li, H.; Grandis, J. R.; Centuori, S.; Johnson, D. E.; Bauman, J. E., Clinical trials optimizing investigator and self-collection of buccal cells for RNA yield. *Laryngoscope Investig Otolaryngol* **2021,** *6* (1), 116-121.
17. Zimmerman, A. W.; Singh, K.; Connors, S. L.; Liu, H.; Panjwani, A. A.; Lee, L. C.; Diggins, E.; Foley, A.; Melnyk, S.; Singh, I. N.; James, S. J.; Frye, R. E.; Fahey, J. W., Randomized controlled trial of sulforaphane and metabolite discovery in children with Autism Spectrum Disorder. *Mol Autism* **2021,** *12* (1), 38.
18. Sivapalan, T.; Melchini, A.; Saha, S.; Needs, P. W.; Traka, M. H.; Tapp, H.; Dainty, J. R.; Mithen, R. F., Bioavailability of Glucoraphanin and Sulforaphane from High-Glucoraphanin Broccoli. *Mol Nutr Food Res* **2018,** *62* (18), e1700911.
19. Bauman, J. E.; Zang, Y.; Sen, M.; Li, C.; Wang, L.; Egner, P. A.; Fahey, J. W.; Normolle, D. P.; Grandis, J. R.; Kensler, T. W.; Johnson, D. E., Prevention of Carcinogen-Induced Oral Cancer by Sulforaphane. *Cancer Prev Res (Phila)* **2016,** *9* (7), 547-57.
20. Doss, J. F.; Jonassaint, J. C.; Garrett, M. E.; Ashley-Koch, A. E.; Telen, M. J.; Chi, J. T., Phase 1 Study of a Sulforaphane-Containing Broccoli Sprout Homogenate for Sickle Cell Disease. *PLoS ONE* **2016,** *11* (4), e0152895.

21. Chartoumpekis, D. V.; Ziros, P. G.; Chen, J. G.; Groopman, J. D.; Kensler, T. W.; Sykiotis, G. P., Broccoli sprout beverage is safe for thyroid hormonal and autoimmune status: Results of a 12-week randomized trial. *Food Chem Toxicol* **2019,** *126*, 1-6.
22. Vinge, F.; Tillqvist, E.; Horwath, O.; Apró, W.; Larsen, F. J.; Sundqvist, M. L., A glucosinolate-rich beverage lowers blood lactate concentrations during submaximal exercise *bioRxiv* **2025**.
23. Flockhart, M.; Nilsson, L. C.; Tillqvist, E. N.; Vinge, F.; Millbert, F.; Lännerström, J.; Nilsson, P. H.; Samyn, D.; Apró, W.; Sundqvist, M. L.; Larsen, F. J., Glucosinolate-rich broccoli sprouts protect against oxidative stress and improve adaptations to intense exercise training. *Redox Biol* **2023,** *67*, 102873.
24. Lopez-Chillon, M. T.; Carazo-Diaz, C.; Prieto-Merino, D.; Zafrilla, P.; Moreno, D. A.; Villano, D., Effects of long-term consumption of broccoli sprouts on inflammatory markers in overweight subjects. *Clin Nutr* **2019,** *38* (2), 745-752.
25. Chen, J. G.; Johnson, J.; Egner, P.; Ng, D.; Zhu, J.; Wang, J. B.; Xue, X. F.; Sun, Y.; Zhang, Y. H.; Lu, L. L.; Chen, Y. S.; Wu, Y.; Zhu, Y. R.; Carmella, S.; Hecht, S.; Jacobson, L.; Munoz, A.; Kensler, K.; Rule, A.; Fahey, J.; Kensler, T.; Groopman, J., Dose-dependent detoxication of the airborne pollutant benzene in a randomized trial of broccoli sprout beverage in Qidong, China. *Am J Clin Nutr* **2019,** *110* (3), 675-684.
26. Fahey, J. W.; Wade, K. L.; Stephenson, K. K.; Panjwani, A. A.; Liu, H.; Cornblatt, G.; Cornblatt, B. S.; Ownby, S. L.; Fuchs, E.; Holtzclaw, W. D.; Cheskin, L. J., Bioavailability of Sulforaphane Following Ingestion of Glucoraphanin-Rich Broccoli Sprout and Seed Extracts with Active Myrosinase: A Pilot Study of the Effects of Proton Pump Inhibitor Administration. *Nutrients* **2019,** *11* (7).
27. Traka, M. H.; Melchini, A.; Coode-Bate, J.; Kadhi, O. A.; Saha, S.; Defernez, M.; Troncoso-Rey, P.; Kibblewhite, H.; O'Neill, C. M.; Bernuzzi, F.; Mythen, L.; Hughes, J.; Needs, P. W.; Dainty, J. R.; Savva, G. M.; Mills, R. D.; Ball, R. Y.; Cooper, C. S.; Mithen, R. F., Transcriptional changes in prostate of men on active surveillance after a 12-mo glucoraphanin-rich broccoli intervention-results from the Effect of Sulforaphane on prostate CAncer PrEvention (ESCAPE) randomized controlled trial. *Am. J. Clin. Nutr.* **2019,** *109* (4), 1133-1144.
28. Sedlak, T. W.; Nucifora, L. G.; Koga, M.; Shaffer, L. S.; Higgs, C.; Tanaka, T.; Wang, A. M.; Coughlin, J. M.; Barker, P. B.; Fahey, J. W.; Sawa, A., Sulforaphane Augments Glutathione and Influences Brain Metabolites in Human Subjects: A Clinical Pilot Study. *Mol Neuropsychiatry* **2018,** *3* (4), 214-222.
29. Tahata, S.; Singh, S. V.; Lin, Y.; Hahm, E. R.; Beumer, J. H.; Christner, S. M.; Rao, U. N.; Sander, C.; Tarhini, A. A.; Tawbi, H.; Ferris, L. K.; Wilson, M.; Rose, A.; Dietz, C. M.; Hughes, E.; Fahey, J. W.; Leachman, S. A.; Cassidy, P. B.; Butterfield, L. H.; Zarour, H. M.; Kirkwood, J. M., Evaluation of Biodistribution of Sulforaphane after Administration of Oral Broccoli Sprout Extract in Melanoma Patients with Multiple Atypical Nevi. *Cancer Prev Res (Phila)* **2018,** *11* (7), 429-438.

30. Bent, S.; Lawton, B.; Warren, T.; Widjaja, F.; Dang, K.; Fahey, J. W.; Cornblatt, B.; Kinchen, J. M.; Delucchi, K.; Hendren, R. L., Identification of urinary metabolites that correlate with clinical improvements in children with autism treated with sulforaphane from broccoli. *Mol Autism* **2018,** *9*, 35.
31. Housley, L.; Magana, A. A.; Hsu, A.; Beaver, L. M.; Wong, C. P.; Stevens, J. F.; Choi, J.; Jiang, Y.; Bella, D.; Williams, D. E.; Maier, C. S.; Shannon, J.; Dashwood, R. H.; Ho, E., Untargeted Metabolomic Screen Reveals Changes in Human Plasma Metabolite Profiles Following Consumption of Fresh Broccoli Sprouts. *Mol Nutr Food Res* **2018,** *62* (19), e1700665.
32. Okunade, O.; Niranjan, K.; Ghawi, S. K.; Kuhnle, G.; Methven, L., Supplementation of the Diet by Exogenous Myrosinase via Mustard Seeds to Increase the Bioavailability of Sulforaphane in Healthy Human Subjects after the Consumption of Cooked Broccoli. *Mol Nutr Food Res* **2018,** *62* (18), e1700980.
33. Axelsson, A. S.; Tubbs, E.; Mecham, B.; Chacko, S.; Nenonen, H. A.; Tang, Y.; Fahey, J. W.; Derry, J. M. J.; Wollheim, C. B.; Wierup, N.; Haymond, M. W.; Friend, S. H.; Mulder, H.; Rosengren, A. H., Sulforaphane reduces hepatic glucose production and improves glucose control in patients with type 2 diabetes. *Sci Transl Med* **2017,** *9* (394).
34. Fahey, J. W.; Wade, K. L.; Wehage, S. L.; Holtzclaw, W. D.; Liu, H.; Talalay, P.; Fuchs, E.; Stephenson, K. K., Stabilized sulforaphane for clinical use: Phytochemical delivery efficiency. *Mol Nutr Food Res* **2017,** *61* (4).
35. Duran, C. G.; Burbank, A. J.; Mills, K. H.; Duckworth, H. R.; Aleman, M. M.; Kesic, M. J.; Peden, D. B.; Pan, Y.; Zhou, H.; Hernandez, M. L., A proof-of-concept clinical study examining the NRF2 activator sulforaphane against neutrophilic airway inflammation. *Respir Res* **2016,** *17* (1), 89.
36. Müller, L.; Meyer, M.; Bauer, R. N.; Zhou, H.; Zhang, H.; Jones, S.; Robinette, C.; Noah, T. L.; Jaspers, I., Effect of Broccoli Sprouts and Live Attenuated Influenza Virus on Peripheral Blood Natural Killer Cells: A Randomized, Double-Blind Study. *PLoS ONE* **2016,** *11* (1), e0147742.
37. Sudini, K.; Diette, G. B.; Breysse, P. N.; McCormack, M. C.; Bull, D.; Biswal, S.; Zhai, S.; Brereton, N.; Peng, R. D.; Matsui, E. C., A Randomized Controlled Trial of the Effect of Broccoli Sprouts on Antioxidant Gene Expression and Airway Inflammation in Asthmatics. *J Allergy Clin Immunol Pract* **2016,** *4* (5), 932-40.
38. Wise, R. A.; Holbrook, J. T.; Criner, G.; Sethi, S.; Rayapudi, S.; Sudini, K. R.; Sugar, E. A.; Burke, A.; Thimmulappa, R.; Singh, A.; Talalay, P.; Fahey, J. W.; Berenson, C. S.; Jacobs, M. R.; Biswal, S.; Leatherman, G.; Daniel, M.; Thurman, A.; Marchetti, N.; Kim, V.; Shenoy, K.; Smith, H.; Rosario, M.; Bolla, S.; Mandapati, C.; Eberhardt, E.; Kruzel, R.; Kumar, S.; Noel, S.; Beselman, A.; Amend-Libercci, D.; Ewing, C.; Hart, A.; Lears, A.; Nowakowski, D.; Prusakowski, N.; Shade, D.; Yasin, R.; Brown, E.; Wang, L.; O'Connor, G.; Strange, C.; Wendt, C.; Punturieri, A.; Viviano, L. W., Lack of effect of oral sulforaphane administration on Nrf2 expression in COPD: A randomized, double-blind, placebo controlled trial. *PLoS ONE* **2016,** *11* (11).

39. Alumkal, J. J.; Slottke, R.; Schwartzman, J.; Cherala, G.; Munar, M.; Graff, J. N.; Beer, T. M.; Ryan, C. W.; Koop, D. R.; Gibbs, A.; Gao, L.; Flamiatos, J. F.; Tucker, E.; Kleinschmidt, R.; Mori, M., A phase II study of sulforaphane-rich broccoli sprout extracts in men with recurrent prostate cancer. *Invest. New Drugs* **2015,** *33* (2), 480-489.
40. Armah, C. N.; Derdemezis, C.; Traka, M. H.; Dainty, J. R.; Doleman, J. F.; Saha, S.; Leung, W.; Potter, J. F.; Lovegrove, J. A.; Mithen, R. F., Diet rich in high glucoraphanin broccoli reduces plasma LDL cholesterol: Evidence from randomised controlled trials. *Mol Nutr Food Res* **2015,** *59* (5), 918-26.
41. Atwell, L. L.; Zhang, Z.; Mori, M.; Farris, P.; Vetto, J. T.; Naik, A. M.; Oh, K. Y.; Thuillier, P.; Ho, E.; Shannon, J., Sulforaphane Bioavailability and Chemopreventive Activity in Women Scheduled for Breast Biopsy. *Cancer Prev Res (Phila)* **2015,** *8* (12), 1184-1191.
42. Atwell, L. L.; Hsu, A.; Wong, C. P.; Stevens, J. F.; Bella, D.; Yu, T. W.; Pereira, C. B.; Löhr, C. V.; Christensen, J. M.; Dashwood, R. H.; Williams, D. E.; Shannon, J.; Ho, E., Absorption and chemopreventive targets of sulforaphane in humans following consumption of broccoli sprouts or a myrosinase-treated broccoli sprout extract. *Mol Nutr Food Res* **2015,** *59* (3), 424-33.
43. Brown, R. H.; Reynolds, C.; Brooker, A.; Talalay, P.; Fahey, J. W., Sulforaphane improves the bronchoprotective response in asthmatics through Nrf2-mediated gene pathways. *Respir Res* **2015,** *16* (1), 106.
44. Chang, Y. W.; Jang, J. Y.; Kim, Y. H.; Kim, J. W.; Shim, J. J., The Effects of Broccoli Sprout Extract Containing Sulforaphane on Lipid Peroxidation and Helicobacter pylori Infection in the Gastric Mucosa. *Gut Liver* **2015,** *9* (4), 486-93.
45. Cipolla, B. G.; Mandron, E.; Lefort, J. M.; Coadou, Y.; Della Negra, E.; Corbel, L.; Le Scodan, R.; Azzouzi, A. R.; Mottet, N., Effect of Sulforaphane in Men with Biochemical Recurrence after Radical Prostatectomy. *Cancer Prev Res (Phila)* **2015,** *8* (8), 712-9.
46. Fahey, J. W.; Holtzclaw, W. D.; Wehage, S. L.; Wade, K. L.; Stephenson, K. K.; Talalay, P., Sulforaphane Bioavailability from Glucoraphanin-Rich Broccoli: Control by Active Endogenous Myrosinase. *PLoS ONE* **2015,** *10* (11), e0140963.
47. Kikuchi, M.; Ushida, Y.; Shiozawa, H.; Umeda, R.; Tsuruya, K.; Aoki, Y.; Suganuma, H.; Nishizaki, Y., Sulforaphane-rich broccoli sprout extract improves hepatic abnormalities in male subjects. *World J Gastroenterol* **2015,** *21* (43), 12457-67.
48. Medina, S.; Domínguez-Perles, R.; Moreno, D. A.; García-Viguera, C.; Ferreres, F.; Gil, J. I.; Gil-Izquierdo, Á., The intake of broccoli sprouts modulates the inflammatory and vascular prostanoids but not the oxidative stress-related isoprostanes in healthy humans. *Food Chem* **2015,** *173*, 1187-94.
49. Shiina, A.; Kanahara, N.; Sasaki, T.; Oda, Y.; Hashimoto, T.; Hasegawa, T.; Yoshida, T.; Iyo, M.; Hashimoto, K., An open study of sulforaphane-rich broccoli sprout extract in patients with schizophrenia. *Clin. Psychopharmacol. Neurosci.* **2015,** *13* (1), 62-67.

50. Ushida, Y.; Suganuma, H.; Yanaka, A., Low-Dose of the Sulforaphane Precursor Glucoraphanin as a Dietary Supplement Induces Chemoprotective Enzymes in Humans. *Food and Nutrition Sciences* **2015,** *6* (17), 1603-1612.
51. Bahadoran, Z.; Golzarand, M.; F, A.; Mirmiran, P., *Complementary and alternative medicinal effects of broccoli sprouts powder on Helicobacter pylori eradication rate in type 2 diabetic patients: A randomized clinical trial*. 2014.
52. Baier, S. R.; Zbasnik, R.; Schlegel, V.; Zempleni, J., Off-target effects of sulforaphane include the derepression of long terminal repeats through histone acetylation events. *J Nutr Biochem* **2014,** *25* (6), 665-8.
53. Egner, P. A.; Chen, J. G.; Zarth, A. T.; Ng, D. K.; Wang, J. B.; Kensler, K. H.; Jacobson, L. P.; Munoz, A.; Johnson, J. L.; Groopman, J. D.; Fahey, J. W.; Talalay, P.; Zhu, J.; Chen, T. Y.; Qian, G. S.; Carmella, S. G.; Hecht, S. S.; Kensler, T. W., Rapid and sustainable detoxication of airborne pollutants by broccoli sprout beverage: results of a randomized clinical trial in China. *Cancer Prev Res (Phila)* **2014,** *7* (8), 813-823.
54. Heber, D.; Li, Z.; Garcia-Lloret, M.; Wong, A. M.; Lee, T. Y.; Thames, G.; Krak, M.; Zhang, Y.; Nel, A., Sulforaphane-rich broccoli sprout extract attenuates nasal allergic response to diesel exhaust particles. *Food and Function* **2014,** *5* (1), 35-41.
55. Noah, T. L.; Zhang, H.; Zhou, H.; Glista-Baker, E.; Müller, L.; Bauer, R. N.; Meyer, M.; Murphy, P. C.; Jones, S.; Letang, B.; Robinette, C.; Jaspers, I., Effect of broccoli sprouts on nasal response to live attenuated influenza virus in smokers: A randomized, double-blind study. *PLoS ONE* **2014,** *9* (6).
56. Singh, K.; Connors, S. L.; Macklin, E. A.; Smith, K. D.; Fahey, J. W.; Talalay, P.; Zimmerman, A. W., Sulforaphane treatment of autism spectrum disorder (ASD). *Proc Natl Acad Sci U S A* **2014,** *111* (43), 15550-5.
57. Lynch, R.; Diggins, E. L.; Connors, S. L.; Zimmerman, A. W.; Singh, K.; Liu, H.; Talalay, P.; Fahey, J. W., Sulforaphane from Broccoli Reduces Symptoms of Autism: A Follow-up Case Series from a Randomized Double-blind Study. *Glob Adv Health Med* **2017,** *6*, 2164957X17735826.
58. Armah, C. N.; Traka, M. H.; Dainty, J. R.; Defernez, M.; Janssens, A.; Leung, W.; Doleman, J. F.; Potter, J. F.; Mithen, R. F., A diet rich in high-glucoraphanin broccoli interacts with genotype to reduce discordance in plasma metabolite profiles by modulating mitochondrial function. *Am J Clin Nutr* **2013,** *98* (3), 712-22.
59. Meyer, M.; Kesic, M. J.; Clarke, J.; Ho, E.; Simmen, R. C.; Diaz-Sanchez, D.; Noah, T. L.; Jaspers, I., Sulforaphane induces SLPI secretion in the nasal mucosa. *Respir Med* **2013,** *107* (3), 472-5.
60. Poulton, E. J.; Levy, L.; Lampe, J. W.; Shen, D. D.; Tracy, J.; Shuhart, M. C.; Thummel, K. E.; Eaton, D. L., Sulforaphane is not an effective antagonist of the human pregnane X-receptor in vivo. *Toxicol Appl Pharmacol* **2013,** *266* (1), 122-31.
61. Bahadoran, Z.; Tohidi, M.; Nazeri, P.; Mehran, M.; Azizi, F.; Mirmiran, P., Effect of broccoli sprouts on insulin resistance in type 2 diabetic patients: a randomized double-blind clinical trial. *Int J Food Sci Nutr* **2012,** *63* (7), 767-71.

62. Cramer, J. M.; Teran-Garcia, M.; Jeffery, E. H., Enhancing sulforaphane absorption and excretion in healthy men through the combined consumption of fresh broccoli sprouts and a glucoraphanin-rich powder. *The British journal of nutrition* **2012,** *107* (9), 1333-1338.
63. Fahey, J. W.; Wehage, S. L.; Holtzclaw, W. D.; Kensler, T. W.; Egner, P. A.; Shapiro, T. A.; Talalay, P., Protection of humans by plant glucosinolates: efficiency of conversion of glucosinolates to isothiocyanates by the gastrointestinal microflora. *Cancer Prev Res (Phila)* **2012,** *5* (4), 603-11.
64. Kensler, T. W.; Ng, D.; Carmella, S. G.; Chen, M.; Jacobson, L. P.; Munoz, A.; Egner, P. A.; Chen, J. G.; Qian, G. S.; Chen, T. Y.; Fahey, J. W.; Talalay, P.; Groopman, J. D.; Yuan, J. M.; Hecht, S. S., Modulation of the metabolism of airborne pollutants by glucoraphanin-rich and sulforaphane-rich broccoli sprout beverages in Qidong, China. *Carcinogenesis* **2012,** *33* (1), 101-7.
65. Mirmiran, P.; Bahadoran, Z.; Hosseinpanah, F.; Keyzad, A.; Azizi, F., Effects of broccoli sprout with high sulforaphane concentration on inflammatory markers in type 2 diabetic patients: A randomized double-blind placebo-controlled clinical trial. *Journal of Functional Foods* **2012,** *4* (4), 837-841.
66. Saha, S.; Hollands, W.; Teucher, B.; Needs, P. W.; Narbad, A.; Ortori, C. A.; Barrett, D. A.; Rossiter, J. T.; Mithen, R. F.; Kroon, P. A., Isothiocyanate concentrations and interconversion of sulforaphane to erucin in human subjects after consumption of commercial frozen broccoli compared to fresh broccoli. *Mol Nutr Food Res* **2012,** *56* (12), 1906-16.
67. Bahadoran, Z.; Mirmiran, P.; Hosseinpanah, F.; Hedayati, M.; Hosseinpour-Niazi, S.; Azizi, F., Broccoli sprouts reduce oxidative stress in type 2 diabetes: a randomized double-blind clinical trial. *Eur J Clin Nutr* **2011,** *65* (8), 972-7.
68. Clarke, J. D.; Hsu, A.; Riedl, K.; Bella, D.; Schwartz, S. J.; Stevens, J. F.; Ho, E., Bioavailability and inter-conversion of sulforaphane and erucin in human subjects consuming broccoli sprouts or broccoli supplement in a cross-over study design. *Pharmacol Res* **2011,** *64* (5), 456-63.
69. Egner, P. A.; Chen, J. G.; Wang, J. B.; Wu, Y.; Sun, Y.; Lu, J. H.; Zhu, J.; Zhang, Y. H.; Chen, Y. S.; Friesen, M. D.; Jacobson, L. P.; Munoz, A.; Ng, D.; Qian, G. S.; Zhu, Y. R.; Chen, T. Y.; Botting, N. P.; Zhang, Q.; Fahey, J. W.; Talalay, P.; Groopman, J. D.; Kensler, T. W., Bioavailability of Sulforaphane from two broccoli sprout beverages: results of a short-term, cross-over clinical trial in Qidong, China. *Cancer Prev Res (Phila)* **2011,** *4* (3), 384-95.
70. Christiansen, B.; Bellostas Muguerza, N.; Petersen, A. M.; Kveiborg, B.; Madsen, C. R.; Thomas, H.; Ihlemann, N.; Sørensen, J. C.; Køber, L.; Sørensen, H.; Torp-Pedersen, C.; Domínguez, H., Ingestion of broccoli sprouts does not improve endothelial function in humans with hypertension. *PLoS ONE* **2010,** *5* (8), e12461.
71. Hanlon, N.; Coldham, N.; Gielbert, A.; Sauer, M. J.; Ioannides, C., Repeated intake of broccoli does not lead to higher plasma levels of sulforaphane in human volunteers. *Cancer Lett* **2009,** *284* (1), 15-20.
72. Riedl, M. A.; Saxon, A.; Diaz-Sanchez, D., Oral sulforaphane increases Phase II antioxidant enzymes in the human upper airway. *Clin Immunol* **2009,** *130* (3), 244-51.

73. Riso, P.; Martini, D.; Visioli, F.; Martinetti, A.; Porrini, M., Effect of broccoli intake on markers related to oxidative stress and cancer risk in healthy smokers and nonsmokers. *Nutr Cancer* **2009,** *61* (2), 232-7.
74. Yanaka, A.; Fahey, J. W.; Fukumoto, A.; Nakayama, M.; Inoue, S.; Zhang, S.; Tauchi, M.; Suzuki, H.; Hyodo, I.; Yamamoto, M., Dietary sulforaphane-rich broccoli sprouts reduce colonization and attenuate gastritis in Helicobacter pylori-infected mice and humans. *Cancer Prev Res (Phila)* **2009,** *2* (4), 353-60.
75. Traka, M.; Gasper, A. V.; Melchini, A.; Bacon, J. R.; Needs, P. W.; Frost, V.; Chantry, A.; Jones, A. M.; Ortori, C. A.; Barrett, D. A.; Ball, R. Y.; Mills, R. D.; Mithen, R. F., Broccoli consumption interacts with GSTM1 to perturb oncogenic signalling pathways in the prostate. *PLoS ONE* **2008,** *3* (7), e2568.
76. Vermeulen, M.; Klöpping-Ketelaars, I. W.; van den Berg, R.; Vaes, W. H., Bioavailability and kinetics of sulforaphane in humans after consumption of cooked versus raw broccoli. *J Agric Food Chem* **2008,** *56* (22), 10505-9.
77. Talalay, P.; Fahey, J. W.; Healy, Z. R.; Wehage, S. L.; Benedict, A. L.; Min, C.; Dinkova-Kostova, A. T., Sulforaphane mobilizes cellular defenses that protect skin against damage by UV radiation. *Proc Natl Acad Sci U S A* **2007,** *104* (44), 17500-5.
78. Cornblatt, B. S.; Ye, L.; Dinkova-Kostova, A. T.; Erb, M.; Fahey, J. W.; Singh, N. K.; Chen, M. S.; Stierer, T.; Garrett-Mayer, E.; Argani, P.; Davidson, N. E.; Talalay, P.; Kensler, T. W.; Visvanathan, K., Preclinical and clinical evaluation of sulforaphane for chemoprevention in the breast. *Carcinogenesis* **2007,** *28* (7), 1485-90.
79. Gasper, A. V.; Traka, M.; Bacon, J. R.; Smith, J. A.; Taylor, M. A.; Hawkey, C. J.; Barrett, D. A.; Mithen, R. F., Consuming Broccoli Does Not Induce Genes Associated with Xenobiotic Metabolism and Cell Cycle Control in Human Gastric Mucosa12. *The Journal of Nutrition* **2007,** *137* (7), 1718-1724.
80. Myzak, M. C.; Tong, P.; Dashwood, W. M.; Dashwood, R. H.; Ho, E., Sulforaphane retards the growth of human PC-3 xenografts and inhibits HDAC activity in human subjects. *Exp Biol Med (Maywood)* **2007,** *232* (2), 227-34.
81. Rungapamestry, V.; Duncan, A. J.; Fuller, Z.; Ratcliffe, B., Effect of meal composition and cooking duration on the fate of sulforaphane following consumption of broccoli by healthy human subjects. *Br J Nutr* **2007,** *97* (4), 644-52.
82. Shapiro, T. A.; Fahey, J. W.; Dinkova-Kostova, A. T.; Holtzclaw, W. D.; Stephenson, K. K.; Wade, K. L.; Ye, L.; Talalay, P., Safety, tolerance, and metabolism of broccoli sprout glucosinolates and isothiocyanates: A clinical phase I study. *Nutr. Cancer* **2006,** *55* (1), 53-62.
83. Gasper, A. V.; Al-Janobi, A.; Smith, J. A.; Bacon, J. R.; Fortun, P.; Atherton, C.; Taylor, M. A.; Hawkey, C. J.; Barrett, D. A.; Mithen, R. F., Glutathione S-transferase M1 polymorphism and metabolism of sulforaphane from standard and high-glucosinolate broccoli. *Am. J. Clin. Nutr.* **2005,** *82* (6), 1283-1291.
84. Kensler, T. W.; Chen, J. G.; Egner, P. A.; Fahey, J. W.; Jacobson, L. P.; Stephenson, K. K.; Ye, L.; Coady, J. L.; Wang, J. B.; Wu, Y.; Sun, Y.; Zhang, Q. N.; Zhang, B. C.; Zhu, Y. R.; Qian, G. S.; Carmella, S. G.; Hecht, S. S.; Benning, L.; Gange, S. J.; Groopman, J. D.; Talalay, P., Effects of glucosinolate-rich broccoli sprouts on urinary levels of

aflatoxin-DNA adducts and phenanthrene tetraols in a randomized clinical trial in He Zuo township, Qidong, People's Republic of China. *Cancer Epidemiol Biomarkers Prev* **2005,** *14* (11 Pt 1), 2605-13.
85. Murashima, M.; Watanabe, S.; Zhuo, X. G.; Uehara, M.; Kurashige, A., Phase 1 study of multiple biomarkers for metabolism and oxidative stress after one-week intake of broccoli sprouts. *BioFactors* **2004,** *22* (1-4), 271-5.
86. Walters, D. G.; Young, P. J.; Agus, C.; Knize, M. G.; Boobis, A. R.; Gooderham, N. J.; Lake, B. G., Cruciferous vegetable consumption alters the metabolism of the dietary carcinogen 2-amino-1-methyl-6-phenylimidazo[4,5-b]pyridine (PhIP) in humans. *Carcinogenesis* **2004,** *25* (9), 1659-69.
87. Galan, M. V.; Kishan, A. A.; Silverman, A. L., Oral broccoli sprouts for the treatment of Helicobacter pylori infection: a preliminary report. *Dig Dis Sci* **2004,** *49* (7-8), 1088-90.
88. Ye, L.; Dinkova-Kostova, A. T.; Wade, K. L.; Zhang, Y.; Shapiro, T. A.; Talalay, P., Quantitative determination of dithiocarbamates in human plasma, serum, erythrocytes and urine: pharmacokinetics of broccoli sprout isothiocyanates in humans. *Clin. Chim. Acta* **2002,** *316* (1), 43-53.
89. Hauder, J.; Winkler, S.; Bub, A.; Rüfer, C. E.; Pignitter, M.; Somoza, V., LC-MS/MS quantification of sulforaphane and indole-3-carbinol metabolites in human plasma and urine after dietary intake of selenium-fortified broccoli. *J. Agric. Food Chem.* **2011,** *59* (15), 8047-8057.
90. Conaway, C. C.; Getahun, S. M.; Liebes, L. L.; Pusateri, D. J.; Topham, D. K.; Botero-Omary, M.; Chung, F. L., Disposition of glucosinolates and sulforaphane in humans after ingestion of steamed and fresh broccoli. *Nutr Cancer* **2000,** *38* (2), 168-78.
91. Shapiro, T. A.; Fahey, J. W.; Wade, K. L.; Stephenson, K. K.; Talalay, P., Human metabolism and excretion of cancer chemoprotective glucosinolates and isothiocyanates of cruciferous vegetables. *Cancer Epidemiol Biomarkers Prev* **1998,** *7* (12), 1091-100.
